# The NAD-brain pharmacokinetic study of NAD augmentation in blood and brain using oral precursor supplementation

**DOI:** 10.1016/j.isci.2026.114764

**Published:** 2026-01-27

**Authors:** Haakon Berven, Magnus Svensen, Heidi Eikeland, Nora Tvedten, Erika V. Sheard, Solveig Amdahl Af Geijerstam, Mona Søgnen, Adrian McCann, Lena Arnsten, Ove Årseth, Vivian Skjeie, Arve Hjellbrekke, Geir-Olve Skeie, Yamila N. Torres Cleuren, Gonzalo S. Nido, Kristoffer Haugarvoll, Frank Riemer, Charalampos Tzoulis, Christian Dölle

**Affiliations:** 1 Neuro-SysMed, Department of Neurology, Haukeland University Hospital, 5021 Bergen, Norway; 2 Department of Clinical Medicine, University of Bergen, 5020 Bergen, Norway; 3 K.G. Jebsen Center for Translational Research in Parkinson’s disease, University of Bergen, 5020 Bergen, Norway; 4 Bevital AS, 5068 Bergen, Norway; 5 Department of Radiology, Haukeland University Hospital, 5020 Bergen, Norway; 6 Mohn Medical Imaging and Visualization Centre (MMIV), Department of Radiology, Haukeland University Hospital, 5020 Bergen, Norway

**Keywords:** Biopharmaceuticals, Health sciences, Pharmaceutical compounds formulation, Pharmaceutical preparation, Pharmaceutical science

## Abstract

Nicotinamide adenine dinucleotide (NAD) augmentation therapy (NAD-AT) is increasingly explored in clinical trials across multiple indications, especially neurological diseases, yet its human pharmacokinetic profile remains incompletely defined. We report findings from a phase I pharmacokinetic trial assessing systemic and cerebral responses to oral NAD precursors in healthy individuals (*n* = 6) and persons with Parkinson’s disease (*n* = 6) receiving 1,200 mg/day nicotinamide riboside or nicotinamide mononucleotide. Blood NAD increased slowly, plateauing after approximately two weeks of treatment, and declined with similarly slow kinetics following treatment discontinuation. Cerebral NAD levels increased measurably after four weeks of treatment. NAD-related metabolites showed faster increase and washout dynamics compared to NAD itself. Collectively, these data suggest that effective NAD-AT requires sustained oral administration over at least 2–4 weeks and that once-daily dosing is sufficient to maintain stable NAD levels. NAD responses exhibited considerable interindividual variability, but were not influenced by disease status or sex, indicating broad applicability.

## Highlights

• Oral NAD-augmentation with NR or NMN requires ∼2 weeks to reach steady-state
• Elevated NAD declines slowly with a half-life of approximately 6.25 days in blood
• The extent of NAD augmentation shows substantial interindividual variation
• The extent of NAD augmentation is independent of sex and Parkinson’s disease status

## Graphical abstract

**Figure:**
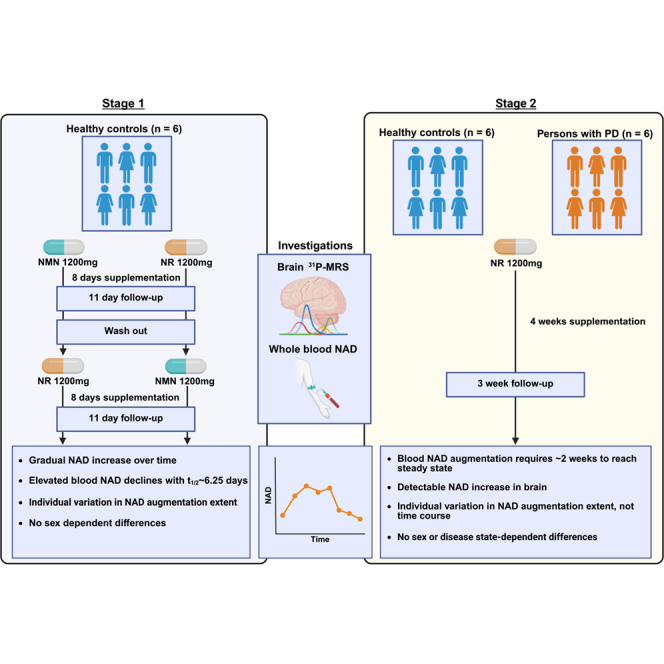

## Introduction

Nicotinamide adenine dinucleotide (NAD) is an essential cofactor for cellular function and survival. Constantly shuttling between its oxidized (NAD^+^) and reduced (NADH) form, NAD plays a pivotal role as an electron carrier in a wide range of redox reactions, including mitochondrial oxidative phosphorylation. Furthermore, NAD^+^ serves as substrate for numerous vital signaling pathways including those involved in DNA repair, energy metabolism, mitochondrial function, inflammation signaling, post-translational modifications (such as deacetylation), and regulation of gene expression.^1^^,^^2^ These signaling reactions consume NAD, necessitating its continuous biosynthesis to maintain cellular levels.^3^

NAD levels have been shown to decline with age in several animal models, and human studies indicate a similar trend.^4^^,^^5^^,^^6^^,^^7^^,^^8^ This decline has been hypothesized to contribute to aging-related pathologies, including neurodegenerative, cardiovascular, and metabolic diseases. Moreover, supplementation with NAD precursors, such as nicotinamide riboside (NR), nicotinamide mononucleotide (NMN), and others, has shown promising effects in a wide range of pre-clinical models reflecting different aspects of these conditions.^2^^,^^9^^,^^10^^,^^11^^,^^12^^,^^13^^,^^14^^,^^15^^,^^16^^,^^17^^,^^18^^,^^19^^,^^20^^,^^21^

Motivated by these findings, NAD augmentation therapy (NAD-AT) using biosynthetic precursors is increasingly being explored in clinical trials targeting a broad spectrum of age-related disease, particularly those affecting neurological function.^22^ Phase I–II clinical trials have investigated NAD-AT for conditions such as Parkinson’s disease (PD), Alzheimer’s disease, mitochondrial disorders, obesity, diabetes, and degenerative eye disorders, yielding largely positive early-phase results.^23^^,^^24^^,^^25^^,^^26^^,^^27^^,^^28^^,^^29^^,^^30^ Currently, over 100 clinical trials of NAD augmentation are registered on ClinicalTrials.gov,^31^ and several more are being planned.

Despite the wealth of preclinical data and early clinical insights, significant knowledge gaps remain concerning the pharmacokinetic (PK) profile of NAD-AT in humans. While prior studies have provided glimpses into the PK properties of some NAD precursors, the picture remains incomplete. One study in healthy individuals reported an increase of NAD levels in peripheral blood mononuclear cells (PBMCs) within a few hours of oral NR supplementation,^32^ and stable NAD levels for 24 h have been observed after several days of NR treatment.^33^ Finally, it was indicated that prolonged NR treatment leads to a stably elevated NAD level in blood.^34^ However, a comprehensive, time-resolved analysis of the NAD response during precursor supplementation and its decline after treatment discontinuation is lacking. Moreover, while brain penetration has been documented by one study in persons with PD (PwPs),^25^ whether this also applies to healthy individuals, and the PK profile of the NAD response in the human brain remains unknown.

Here, we investigate the PK profile of NAD augmentation in both healthy individuals and PwPs in blood and brain. Using two different NAD precursors, NR and NMN, we show that the NAD response to oral supplementation is slow and varies among individuals in extent and maximum NAD level, but interestingly not in the overall time course.

## Results

### Short-term NAD precursor supplementation reveals slow pharmacokinetic response in blood and brain

**Figure 1:**
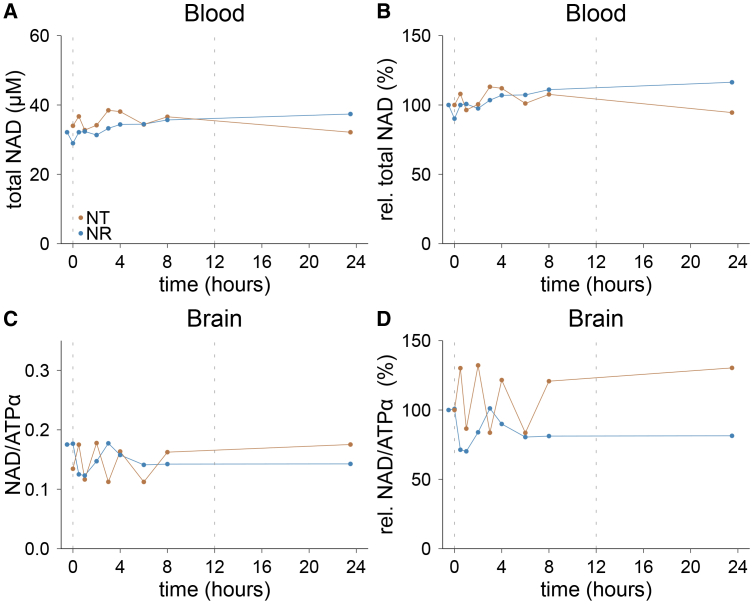
Variation of whole blood and cerebral NAD levels over 24 h with and without oral NR supplementation One healthy male individual received 600 mg NR twice daily (NR) or not (NT) and was monitored over 24 h. (A) Total NAD in whole blood. (B) Relative NAD levels in whole blood compared to baseline. (C) Total NAD in the brain. (D) Relative NAD levels in brain compared to baseline. The dashed lines indicate the time points of oral NR uptake during the treatment day. NT, no treatment; NR, nicotinamide riboside; total NAD, total nicotinamide adenine dinucleotide; NAD/ATPα, total NAD normalized to the alpha-phosphate signal of ATP; ATP, adenosine triphosphate.

We first conducted a pilot experiment with one healthy male individual, to gain insight into the fluctuation of measured blood and brain NAD levels both physiologically and following the intake of an NAD precursor, each during a 24-h period, and to guide the study design and time frame. The participant underwent blood sampling and ^31^P-MRS scanning at nine time points during a 24-h period of either no treatment or treatment with 600 mg NR at time points 0 and 12 h. This design was partly based on previous reports that indicated a response of NAD augmentation to oral NR supplementation within hours.^32^ NAD levels in whole blood (determined by LC-MS/MS analysis), and brain (determined by ^31^P-MRS) showed minor variation during the day with no treatment (Figure 1). Interestingly, NR treatment did not lead to a detectable increase in blood or brain NAD levels during the 24-h period. These findings indicated that 24 h may be too short to detect changes in blood and brain NAD levels induced by oral NR intake, and to establish a PK profile. We therefore adjusted our study schedule to monitor NAD changes over longer time periods in two stages.

In the first stage, using an open label, cross-over design, treatment with 1,200 mg NR or 1,200 mg NMN per day was monitored in 6 healthy individuals (three men and three women, Table 1, Figure S1A) over 19 days, starting with 8 days of precursor supplementation, and followed by a washout period of 11 days. Blood samples and ^31^P-MRS scans were taken once daily for the first three days of each period (i.e., supplementation and washout period), and with greater time intervals toward the end of each period.

**Table 1:** Demographics of participants in NAD-brain stages 1 and 2

Stage 1				
Demographic	All (*n* = 6)	Female (*n* = 3)	Male (*n* = 3)	*p*-value^a^
Age in years	45.00 ± 1.67 Range 43–47	45.33 ± 2.08	44.66 ± 1.52	0.67
BMI in kg/m^2^	24.31 ± 2.08	24.28 ± 2.20	24.34 ± 2.44	0.97
Dose NAD precursor/BW in mg/kg	15.63 ± 2.33	16.32 ± 0.94	14.95 ± 3.35	0.55

Stage 2				
Demographic	All (*n* = 12) 6 M, 6 F	HC (*n* = 6) 3 M, 3 F	PwPs (*n* = 6) 3 M, 3 F	*p*-value^a^
Age in years	54.91 ± 12.14 Range 44–78	46 ± 1.67 Range 44–48	63.83 ± 11.44 Range 45–78	0.01
BMI kg/m^2^	25.08 ± 2.60	25.46 ± 2.71	24.70 ± 2.67	0.63
Dose NAD precursor/BW in mg/kg	15.84 ± 2.33	15.53 ± 2.36	16.14 ± 2.47	0.66

Values are presented as mean ± standard deviation. BMI, body mass index; BW, body weight; M, male; F, female; NAD, nicotinamide adenine dinucleotide; HC, healthy controls; PwPs, persons with Parkinson’s disease.

a *p* values represent comparisons between groups (i.e., male vs. female in stage 1 and HC vs. PwPs in stage 2). *p* values were calculated using independent two-sided t-tests. Data are shown in aggregate to comply with data protection laws.

**Figure 2:**
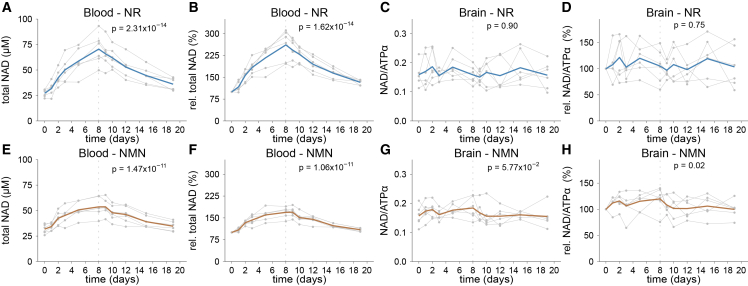
Change in blood and brain NAD levels in stage 1 of the NAD-brain study (A–F) Six healthy individuals were monitored over 19 days and received 600 mg NR (A–D) or NMN (E–H) twice daily for the first 8 days. (A–E) Individual (gray) and mean (blue, brown) total NAD levels in whole blood. (B–F) Individual (gray) and mean (blue, brown) relative total NAD levels in whole blood compared to baseline. (C–G) Individual (gray) and mean (blue, brown) total NAD levels in brain. (D–H) Individual (gray) and mean (blue, brown) relative total NAD levels in brain compared to baseline. The dashed line indicates the last time point of oral NR or NMN supplementation. *p* values represent statistical testing between baseline and the time point indicated by the dashed line. All *p* values were calculated using a linear mixed model where the dependent variable was metabolite level as a function of time with individual participants as random effects. Total NAD, total nicotinamide adenine dinucleotide; NAD/ATPα, total NAD normalized to the alpha-phosphate signal of ATP; ATP, adenosine triphosphate; NR, nicotinamide riboside; NMN, nicotinamide mononucleotide.

Both NR and NMN showed excellent tolerability with no moderate or severe adverse events observed during this stage (Table S1). Upon NR supplementation, blood NAD levels increased throughout the 8-day supplementation period, from 27.3 ± 3.65 μM to 70.51 ± 15.32 μM (LME, *p* = 2.31 x 10^−14^; Figure 2A), corresponding to a mean change from baseline of 161.17 ± 51.26% (Figure 2B). Notably, there was considerable interindividual variation, with individual NAD responses ranging from 96.46% to 211.25% increase from baseline on day 8. Following discontinuation of NR supplementation, blood NAD levels declined gradually during the washout period (LME, *p* = 7.46x10^−15^). However, not all participants reached their respective baseline levels within the 11-day washout period. By day 19 the mean NAD level remained slightly but statistically significantly elevated at 36.10 ± 6.05 μM, corresponding to a 32.74 ± 8.65% increase from baseline (paired *t* test, *p* = 6.18x10^−4^).

Cerebral NAD levels, monitored by ^31^P-MRS, showed no significant increase at the group level over time (LME, *p* = 0.90, Figure 2C) up to day 8, when blood NAD levels were at their peak. The change in measured NAD levels exhibited high interindividual variation, ranging from −39.91% to 48.93% on day 8 compared to baseline (Figure 2D).

Supplementation with NMN resulted in similar changes of blood NAD levels over time. Blood NAD concentrations rose with a rate similar to NR, from 31.79 ± 4.47 μM to 53.67 ± 9.41 μM on day 8 (LME, *p* = 1.47x10^−11^, Figure 2E), corresponding to a mean increase from baseline of 68.87 ± 18.37%, ranging from 38.06% to 89.57% (Figure 2F). Individual responses showed a moderate correlation between NR and NMN (Spearman r = 0.57, *p* = 9.90x10^−3^). Unlike NR, elevated NAD levels remained stable for circa 24 h after the cessation of NMN treatment, peaking on day 9 at 53.71 ± 8.14 μM, representing a 69.44 ± 17.32% increase from baseline (LME, *p* = 1.04x10^−13^, Figure 2F). As with NR supplementation, NAD levels remained slightly elevated at the end of the washout period at 34.69 ± 4.77 μM, corresponding to an increase of 9.30 ± 5.35% compared to baseline (paired *t* test, *p* = 7.37x10^−3^).

Like NR, NMN supplementation did not lead to a significant detectable change in brain NAD levels over time, nor at the end of supplementation on day 8 (LME, *p* = 5.57x10^−2^), or on day 9 (LME, *p* = 0.24), when blood NAD levels were at their peak (Figure 2G). Considerable interindividual variation was seen, with the change from baseline to day 8 ranging from −24.25% to 40.32% (Figure 2H).

A weak correlation between brain and blood NAD levels was seen in the NMN cohort, when considering relative increase from baseline (Figure S2D), but not with NR (Figures S2A and S2B).

A comparison between the two NAD precursors revealed that NR produced an about 2.3-fold higher increase in blood NAD levels compared to NMN up to day 8 (LME, *p* = 1.25x10^−6^). This difference remained significant after adjusting for the compounds’ molecular weights (NRCl, 290.70 g/mol, NMN, 334.22 g/mol, corresponding to ∼15% more NR molecules per dose), and after aligning to each group’s time point of maximal NAD increase (day 8 for NR, day 9 for NMN, LME *p* = 1.17x10^−7^). In contrast, brain NAD measurements did not show a difference between NR and NMN (day 8 LME, *p* = 0.26; day 8 [NR] and day 9 [NMN] LME, *p* = 0.50).

In blood, both oxidized (NAD^+^) and reduced (NADH) forms increased in parallel with total NAD (Figures S3A–S3H). The NAD^+^/NADH ratio also rose significantly from 34.79 ± 9.47 to 44.27 6.80 on day 8 for NR (LME, *p* = 6.61x10^−4^), reaching a maximum on day 9 at 49.14 ± 9.39. For NMN, the ratio increased from 32.88 ± 8.43 to 40.31 ± 4.39 on day 8 (LME, *p* = 1.28x10^−3^) reaching a maximum on day 10 of 41.12 ± 5.30 (Figures S3I–S3L).

To further characterize NAD kinetics, we estimated the half-life of the elevated total NAD levels during the washout period, assuming first order (exponential) decay. Following NR supplementation, NAD levels declined from their day 8 peak with an estimated half-life of 6.76 days (Figure S4). After NMN supplementation, the NAD half-life was similar, at 5.74 days (day 9–19 interval). On average, this corresponded to a mean NAD half-life of ∼6.25 days, consistent with the visual inspection of decay curves. It should be noted that NAD degradation generates nicotinamide, which can be recycled into NAD. Thus, while the exponential model provides an adequate approximation, it may oversimplify the real-life dynamics of NAD metabolism.

### Extended NAD precursor supplementation establishes stably elevated NAD levels in blood and brain of healthy individuals and PwPs

In the first stage of our study, treatment over eight days was not sufficient to reach stably elevated NAD levels in blood, and brain NAD levels remained unchanged. We therefore conducted a second stage with extended treatment of four weeks, similar to our previous trials,^24^^,^^25^ followed by a longer washout period of three weeks. As NR and NMN showed comparable pharmacokinetic profiles, but NR produced a greater NAD increase, only NR was investigated further to reduce participant burden. To assess potential disease-related differences, we included 6 healthy individuals and 6 PwPs (Table 1, Figure S1B). Again, NR treatment showed excellent tolerability, with no moderate or severe adverse events attributed to the treatment (Table S1).

**Figure 3:**
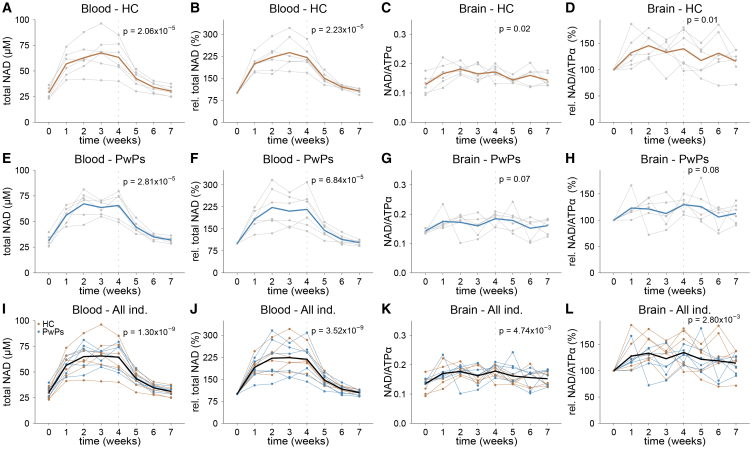
Change in blood and brain NAD levels in stage 2 of the NAD-brain study (A–H) Six healthy individuals (A–D) and six persons with PD (E–H) were monitored over 7 weeks, receiving 600 mg NR twice daily for the first 4 weeks. (A and E) Individual (gray) and mean (blue, brown) total NAD levels in whole blood. (B and F) Individual (gray) and mean (blue, brown) relative total NAD levels in whole blood compared to baseline. (C and G) Individual (gray) and mean (blue, brown) total NAD levels in brain. (D and H) Individual (gray) and mean (blue, brown) relative total NAD levels in brain compared to baseline. (I) Individual (blue, brown) and mean (black) total NAD levels in whole blood from all individuals. (J) Individual (blue, brown) and mean (black) relative total NAD levels in whole blood from all individuals compared to baseline. (K) Individual (blue, brown) and mean (black) total NAD levels in brain. (L) Individual (blue, brown) and mean (black) relative total NAD levels in brain compared to baseline. The dashed line indicates the last time point of oral NR supplementation. *p* values represent statistical testing between baseline and the timepoint indicated by the dashed line. All *p* values were calculated using a linear mixed model where the dependent variable was metabolite level as a function of time with individual participants as random effects. In (I–L), all individuals were compared as one group (*n* = 12). HC, healthy control; PwPs, persons with Parkinson’s disease; Ind., individuals; total NAD, total nicotinamide adenine dinucleotide; NAD/ATPα, total NAD normalized to the alpha-phosphate signal of ATP; ATP, adenosine triphosphate.

In healthy individuals, NR induced a gradual increase in mean blood NAD levels over time (LME, *p* = 2.06x10^−5^), which stabilized after approximately 2 weeks, yielding a new steady state of elevated NAD levels (Figure 3A). Mean NAD levels rose from 28.59 ± 5.44 μM at baseline to 63.25 ± 16.47 μM after 4 weeks of treatment, which corresponded to a 120.65 ± 38.83% increase from baseline (paired *t* test, *p* = 1.19x10^−3^, Figure 3B). During the washout period, NAD levels gradually declined (LME, *p* = 5.56x10^−7^), with most participants returning to within 25% of their baseline levels by week 6 (two weeks of washout), and within 15% of baseline levels by week 7 (three weeks of washout; paired *t* test, *p* = 0.13, Figure 3B).

PwPs exhibited a highly similar profile to the healthy individuals with progressively increasing blood NAD levels over time (LME, *p* = 2.81x10^−5^), stabilizing after two weeks. Their mean NAD levels after 4 weeks of treatment reached 65.62 ± 12.62 μM, corresponding to a 116.16 ± 62.72% increase compared to baseline (paired *t* test, *p* = 2.30x10^−3^; Figures 3E and 3F). During the washout period, most PwPs’ blood NAD levels returned to within 15% of baseline by week 6 (two weeks of washout; paired *t* test, *p* = 0.09).

There were no differences between healthy individuals and PwPs with regard to baseline blood NAD levels (*t* test, *p* = 0.40), NAD response to NR supplementation (LME, *p* = 0.85) or return to baseline during washout (LME, *p* = 0.86). Interindividual variability remained substantial, with 4-week increases ranging from 69.32%–184.61% in controls (Figure 3B) and from 42.11%–209.30% in PwPs (Figure 3F).

The redox ratio of NAD^+^/NADH rose in parallel with total NAD (Figures S3M–S3X), from 38.64 ± 7.41 to 56.29 ± 8.84 in the HC group (LME, *p* = 2.45x10^−3^) and from 35.08 ± 7.83 to 61.00 ± 16.88 in the PwPs group (LME, *p* = 6.40x10^−4^) during the 4 weeks of treatment. During washout it gradually decreased to baseline after approximately 6 weeks for both groups (LME; HC, *p* = 1.29x10^−5^, PwPs, 8.55x10^−6^). No difference was seen in the NAD^+^/NADH ratio between the HC and PwPs groups after 4 weeks (LME, *p* = 0.28).

In the brain, NR supplementation increased NAD levels over time in healthy individuals (LME, *p* = 0.02), stabilizing after two weeks and reaching 39.73 ± 36.22% above baseline after four weeks of treatment (paired *t* test, *p* = 5.77x10^−2^; Figures 3C and 3D). Levels gradually declined during washout to within 15% of baseline (LME, *p* = 0.14). PwPs showed a comparable trajectory, with a non-significant trend in LME analysis (*p* = 0.07) but a significant 29.38 ± 14.80% increase at 4 weeks (paired *t* test, *p* = 0.01; Figures 3G and 3H). There was no significant difference between the groups at baseline (*t* test, *p* = 0.32), or in treatment response (*t* test, *p* = 0.38). Interindividual variability was substantial in both groups, with the change from baseline to week 4 varying from −2.50% to 80.23% in healthy individuals, and 9.96%–50.56% in PwPs (Figures 3D and 3H).

Given the comparable trajectories in both groups, data were combined for secondary analysis (Figures 3I–3L). In the pooled cohort, blood NAD levels increased significantly over time during treatment (LME, *p* = 1.30x10^−9^) and a decline during washout (LME, *p* = 6.51x10^−13^). Mean blood NAD levels rose from 29.94 ± 5.31 to 64.43 ± 14.04 μM after four weeks of treatment, corresponding to a 118.40 ± 49.79% increase compared to baseline (paired *t* test, *p* = 1.94x10^−6^; Figures 3I and 3J), and returned to baseline levels on week 7 (paired *t* test = 0.11).

Brain NAD levels also showed a significant increase during treatment (LME, *p* = 4.74x10^−3^), followed by a decrease during washout (LME, *p* = 0.02). Mean brain NAD levels rose to 134.55 ± 26.93% of baseline after four weeks of treatment (paired *t* test, *p* = 3.05x10^−3^; Figures 3K and 3L) and returned to baseline levels on week 6 (*p* = 0.15). In stage 2, a weak correlation was detected between blood and brain NAD levels (Figures S2E–S2H).

All changes in blood NAD levels were corroborated by an independent analysis using the NADMed method,^35^ which showed similar results in the pilot, stage 1 and stage 2 samples with a high degree of correlation between the two methods (Figures S5 and S6, see STAR Methods for details).

### NADP metabolism is affected by NAD precursor supplementation

Given prior evidence that NAD precursors can affect NADP metabolism,^24^ we also measured the blood NADP pool. In stage 1, NR modestly increased total NADP, NADP^+^ and the NADP^+^/NADPH ratio (Figures S7A, S7B, S7E, S7F, S8A, and S8B), whereas NMN had no effect (Figures S7C, S7D, S7G, S7H, S8C, and S8D). In stage 2, no significant changes were detected in the total NADP pool, and only a minor change in the NADP^+^/NADPH ratio due to minor decrease of NADPH in the PwPs group (Figures S7 and S8).

### Blood and brain NAD levels show high interindividual, but only moderate intraindividual variability

Across both study stages, we observed marked interindividual variability in baseline blood and brain NAD levels, as well as in the magnitude of NAD increase by precursor treatment. To quantify how much of this variation reflected true differences between individuals rather than measurement noise or short-term fluctuations, we calculated intraclass correlations (ICCs) using repeated measures available from the same individuals over time. Specifically, repeat baseline measurements of blood and brain NAD levels were available from five healthy individuals at three different time points, two of which were spaced four weeks apart during stage 1, and the third 45 weeks later, at the start of stage 2.

For blood NAD, baseline measurements showed moderate variability, with interindividual variability accounting for 70.74% and intraindividual differences accounting for only 29.25% of the total variance (ICC = 0.50, *p* = 0.04, Table 2). Brain NAD showed a similar pattern, with interindividual variability explaining 64.64% of the total variance, and intraindividual variability 35.35% of the variance (ICC = 0.52, *p* = 0.03).

**Table 2:** Inter- and intra-individual variation in NAD baseline and NAD-AT response

	Intraclass correlation (95% CI)^a^	*p* value^a^	Intraindividual variance (%)^b^	Interindividual variance (%)^b^
**Blood total NAD (*n* = 5)**				

Baseline	0.50 (−0.06, 0.92)	0.04	29.25	70.74
Augmentation	0.37 (−0.17, 0.88)	0.10	35.26	64.73

**Brain NAD (*n* = 5)**				

Baseline	0.52 (−0.05, 0.92)	0.03	35.35	64.64
Augmentation	0.75 (−0.14, 0.97)	0.04	17.47	82.52

NAD, nicotinamide adenine dinucleotide; NAD-AT, NAD augmentation therapy.

a Intraclass correlation (ICC) and corresponding confidence intervals (CIs) and *p*-values were calculated based on a single rating, consistency-agreement, 2-way mixed-effects model.

b Intraindividual and interindividual variance are presented as percentage of total variance. “Baseline” analysis includes all three values at baseline from stage 1 and 2. “Augmentation” was calculated from day 8 of NR supplementation in stage 1 and week 1 of NR supplementation in stage 2.

Treatment-induced brain NAD increases displayed even higher between-subject consistency, with interindividual differences explaining 82.52% of total variance and intraindividual variability only 17.47% (ICC = 0.75, *p* = 0.04). In contrast, treatment-induced blood NAD changes were more variable (ICC = 0.37, *p* = 0.10), with inter- and intra-individual components contributing 64.73% and 35.26% of the total variance, respectively. Inter- and intraindividual variances and ICCs are summarized in Table 2.

### Interindividual differences in NAD-AT response are sex-independent

**Figure 4:**
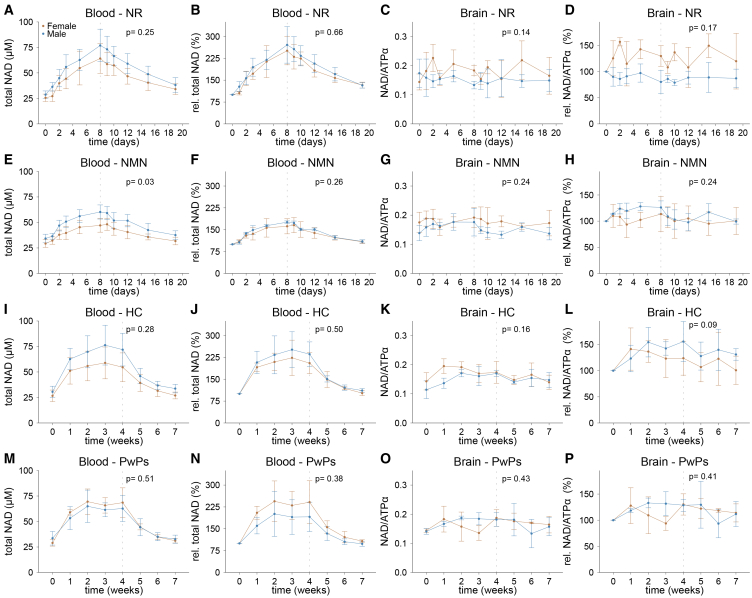
Elevation of whole blood and cerebral NAD levels is independent of disease state and sex Average data from healthy individuals in stage 1 after oral NR (A–D) or NMN supplementation (E–H), and healthy individuals (I–L) and persons with Parkinson’s disease (M–P) in stage 2 separated by sex (brown, female; blue, male). (A, E, I, and M) Mean total NAD levels in whole blood. (B, F, J, and N) Mean relative total NAD levels in whole blood compared to baseline. (C, G, K, and O) Mean total NAD levels in brain. (D, H, L, and P) Mean relative total NAD levels in brain compared to baseline. Data presented as mean ± standard deviation, *n* = 3 per sex. The dashed line indicates the last time point of oral NR or NMN supplementation. *p* values represent statistical testing between male and female groups at the indicated time points. All *p* values were calculated using a linear mixed model where the dependent variable was metabolite level as a function of the interaction of time with sex with individual participants as random effects. Total NAD, total nicotinamide adenine dinucleotide; NAD/ATPα, total NAD normalized to the alpha-phosphate signal of ATP; ATP, adenosine triphosphate; NR, NR treatment; NMN, NMN treatment; HC, healthy controls; PwPs, persons with Parkinson’s disease.

Since males have been shown to have higher blood NAD levels than females,^36^ we assessed whether sex influenced the NAD response upon treatment. Healthy males had slightly higher baseline blood NAD levels, but this did not reach statistical significance (Figures 4A, 4E, and 4I). Moreover, blood NAD levels increased to a higher absolute concentration in males during NMN treatment in stage 1, (LME, *p* = 0.03; Figure 4E), however, the relative increase did not differ between the sexes (LME, *p* = 0.26; Figure 4F). Furthermore, no difference was seen between the sexes with NR supplementation in both healthy individuals and PwPs (Figures 4A, 4I, and 4M). In the brain, no difference in cerebral NAD increase was seen in either healthy individuals or PwPs (Figures 4C, 4G, 4K, and 4O). These results indicate that oral NR and NMN can be dosed the same in males and females.

### NAD-metabolites respond rapidly to initiation and cessation of precursor treatment

We next examined how NAD-related metabolites respond to precursor supplementation. Metabolites consistently detected in all blood samples included nicotinamide (Nam) and its degradation products methyl-nicotinamide (Me-Nam), N-methyl-2-pyridone-5-carboxamide (Me-2-PY), and nicotinamide mononucleotide (NMN). Nicotinic acid riboside (NAR) and nicotinic acid adenine dinucleotide (NAAD), the acid form and direct precursor of NAD in the Preiss-Handler pathway from nicotinic acid,^37^ as well as the Nam degradation product nicotinamide N-oxide (Nam N-oxide), were detectable in most samples taken during precursor supplementation, but were mostly below the limit of detection at baseline and end of the washout period.

**Figure 5:**
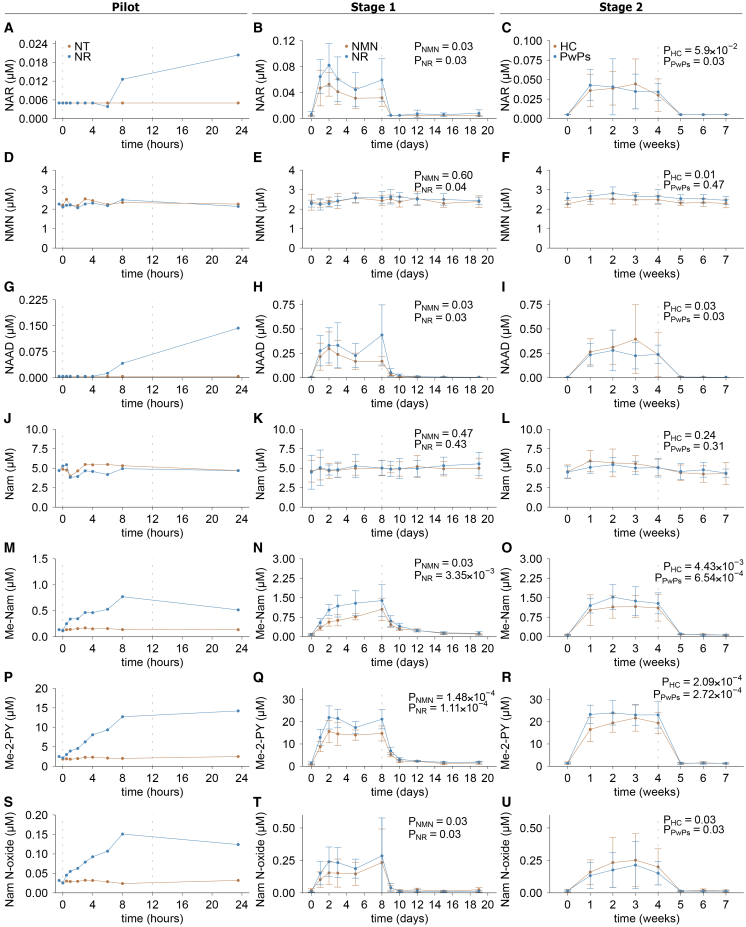
Changes in NAD-related metabolites during the pilot, stage 1 and 2 of the NAD-brain study Plots show data from the pilot without (brown) and with (blue) NR supplementation, mean data after oral supplementation of NR (blue) or NMN (brown) from stage 1 and mean data from healthy individuals (brown) or persons with Parkinson’s disease (blue) after oral NR supplementation from stage 2. The plots show the changes over time of NAR (A–C), NMN (D–F), NAAD (G–I), Nam (J–L), Me-Nam (M–O), Me-2-PY (P–R), and Nam N-oxide (S–U). Stage 1 and stage 2 data (second and third column) presented as mean ± standard deviation. The dashed lines indicate the last timepoint of oral NR or NMN supplementation. *p* values represent statistical testing within groups. All *p* values were calculated using paired two-sided *t* test or Wilcoxon tests between baseline and the last day of supplementation. NR, nicotinamide riboside; NMN, nicotinamide mononucleotide; HC, healthy control; PwPs, persons with Parkinson’s disease; NAR, nicotinic acid riboside; NAAD, nicotinic acid adenine dinucleotide; Nam, nicotinamide; Me-Nam, 1-methyl nicotinamide; Me-2-PY, N1-methyl-2-pyridone-5-carboxamide; Nam N-oxide, nicotinamide N-oxide.

During the pilot experiment, Me-Nam, Me-2-PY, and Nam N-oxide increased within 30 min of oral NR uptake (Figure 5, left panels). NAR and NAAD became detectable by 6 h, while Nam and NMN remained stable throughout the 24-h period. Fluctuation of all metabolites during the placebo-day was minimal.

In both stage 1 and stage 2, these findings were confirmed. Upon NR and NMN supplementation in stage 1, NAAD rose earlier and more rapidly than NAD itself, increasing significantly by day 1 (paired test; NR, *p* = 0.03, NMN, *p* = 0.03), reaching a plateau after ∼5 days and returning to baseline after treatment cessation on day 10 (*p* = 0.1 and *p* = 0.2, respectively; Figure 5H). In stage 2, NAAD levels increased significantly in both healthy individuals and PwPs during week 1 (*p* = 0.03 for both) and normalized one week after supplementation ended (*p* = 1.0; Figure 5I). Metabolites NAR, Me-Nam, and Me-2-PY responded similarly, stabilizing on day 2 and returning to baseline 7–9 days after treatments cessation (Figure 5). NMN levels increased moderately with NR treatment. In stage 1, NMN rose by 13.41 ± 12.53% on day 8 (*p* = 0.09) and by 15.25 ± 5.86% on day 10 (*p* = 0.03; Figure 5E), but did not change with NMN supplementation. In stage 2, NMN increased by 9.73 ± 6.97% in healthy controls (*p* = 0.03) and remained unchanged in PwPs (Figure 5F). Nam levels were stable throughout all study stages (Figures 5K–5L).

Individual participant trajectories and relative changes for all metabolites are provided in Figures S9–S12. Collectively, these data demonstrate that the NAD metabolome responds swiftly and reversibly to both the initiation and cessation of precursor supplementation, with distinct kinetic profiles across pathway intermediates and degradation products.

## Discussion

The NAD-brain trial provides, to our knowledge, the first systematic characterization of the complete PK profile of NAD augmentation with oral biosynthetic precursors in both blood and brain of healthy individuals and PwPs. We demonstrate that oral NR and NMN produce a gradual increase in blood and brain NAD levels that plateau only after approximately two weeks of supplementation, highlighting a substantially slower kinetic profile in humans compared to that suggested by pre-clinical models.

Previous human studies have provided mostly short-term assessments of NAD kinetics. For example, Trammell et al. reported a ∼47% increase of NAD in PBMCs within 24 h of a single 1,000 mg NR dose,^32^ whereas we observed only ∼15% after 24 h in whole blood. This difference likely reflects both tissue-specific dynamics (i.e., whole blood vs. PBMCs) and methodological differences. We applied LC-MS/MS analysis for the detection of the NAD metabolome, and corroborated NAD^+^ quantification by the NADmed method. Both approaches yielded similar results and correlated greatly, although generally a slightly higher NAD^+^ level was detected by the LC-MS/MS approach. Our results, replicated across health and disease, demonstrate that, in humans, blood and brain NAD levels rise slowly and progressively during oral NR or NMN supplementation, stabilizing only after ∼2 weeks. This is consistent with prior clinical data showing gradual elevation over the first two weeks of NR supplementation.^34^

In contrast, preclinical studies in mice have shown rapid NAD increases in blood within hours of oral NR administration.^32^^,^^38^ Our findings reveal a substantially slower human PK/PD profile, likely reflecting interspecies differences in metabolism and/or gut microbiome composition.^39^ These observations caution against direct extrapolation of rodent NAD precursor kinetics to humans.

After steady state was achieved, NAD levels remained stable throughout treatment, consistent with previous reports of sustained NAD elevation after 2–8 weeks of NR administration.^34^ After treatment cessation, blood and brain NAD levels declined with similarly slow kinetics (half-life ∼6.25 days), requiring 2–3 weeks to return to baseline. This is intriguing, as NAD-degrading signaling reactions are expected to be elevated due to the increased substrate availability. Earlier metabolomic analyses have indeed shown increased levels of nicotinamide degradation intermediates such as Me-Nam and Me-2-PY in several tissues upon NAD precursor supplementation, supporting this notion.^25^^,^^32^^,^^34^^,^^40^ On the other hand, an elevated use of NAD^+^ also increases the main substrate of human NAD synthesis, nicotinamide, which is available for recycling and may thus contribute to an extended maintenance of the elevated NAD levels.^24^^,^^34^^,^^40^ Thus, it is plausible that the delayed return of NAD levels to baseline may reflect ongoing nicotinamide recycling through the salvage pathway. These findings imply that once per day dosing is sufficient to maintain elevated NAD levels and that brief interruptions in treatment have minimal impact on blood and brain NAD levels, once a steady state has been achieved.

Cerebral NAD quantification by ^31^P-MRS at 3T was less sensitive than blood assays, leading to high variability and weak blood-brain correlations. This primarily reflects technical limitations, including large voxel size and imperfect reproducibility of voxel placement. Higher spatial resolution (e.g., finer voxel grids or 7T scanners) could improve measurement sensitivity. Indeed, a recent 7T study detected ∼16% increases in brain NAD after NR supplementation using downfield ^1^H MR spectroscopy.^41^ However, such high-field instruments are rarely available in typical hospital settings. Our ongoing NADAPT trial^42^ applies refined voxel sampling to address these limitations. The ability to reliably quantify brain NAD *in vivo* will be essential for linking peripheral NAD kinetics to central metabolic and functional outcomes.

We observed pronounced interindividual variability in both baseline NAD levels and the NAD response to NAD-AT. In contrast, within-subjects variation remained remarkably lower over a 50-week period. These findings indicate that physiological blood and brain NAD levels, as well as NAD-AT treatment responses, are strongly influenced by individual-specific factors. Genetic predisposition affecting enzymes involved in NAD biosynthesis and metabolism may impact the individual’s capacity of NAD synthesis and degradation. Mutations in genes encoding NAD biosynthetic enzymes such as nicotinamide mononucleotide adenylyl transferase and NAD synthase have been shown to strongly affect overall NAD biosynthesis.^43^^,^^44^ While such pathogenic mutations leading to overt disease can be excluded in our cohort, genetic variants that subtly alter substrate affinity, protein stability and others functional parameters, may cumulatively define an individual’s overall NAD biosynthetic activity. Furthermore, the gut microbiome plays a crucial role on the utilization of oral NAD precursors.^38^^,^^45^ Variations in the microbiome composition of our participants could significantly affect precursor bioavailability, and modulate the interplay between host and microbiome, further contributing to the observed variability.

Regardless of the underlying mechanisms, these results indicate that the NAD-AT regimen may have to be tailored to achieve a specific target level of NAD augmentation. Until the exact factors determining this response are identified, this could potentially be achieved by adjusting the dose based on blood and/or brain NAD response after 2–4 weeks of treatment. While the relationship between precursor dose and blood or brain NAD response is yet unknown, this is currently being tested in our ongoing dose-escalation trial, N-DOSE (Clinicaltrials.gov: NCT05589766).^46^

Sex did not influence the NAD-AT-induced NAD response, indicating that similar regimens of NAD-AT can be applied for men and women. Moreover, no differences were seen in NAD basal levels or NAD-AT response between healthy controls and PwPs. These findings suggest that NAD levels in blood and brain are not altered in PD, and that the disease does not limit NAD synthesis when excess substrate is made available. However, assessment of a larger number of PwPs and controls is required to validate these observations.

The modest NADP response aligns with prior findings from NR-SAFE trial^24^ and likely reflects the lower NR dose employed here. Nevertheless, the NADP pool supports antioxidant pathways central to neuroprotection, suggesting that NADP metabolism warrants greater attention in NAD-therapeutic research, especially at higher dosing.^47^^,^^48^

NR and NMN-mediated NAD augmentation followed similar temporal kinetics, implying a shared metabolic pathway, but NR produced a significantly greater amplitude of response, which persisted after correction for higher molecule number delivery. This difference may stem from distinct pharmacological properties, including intestinal uptake and food-dependent absorption. Extending NMN treatment beyond 8 days may yield comparable NAD levels, but head-to-head trials with harmonized dosing and controlled meal timing will be required to determine their relative bioavailability and efficacy.

**Figure 6:**
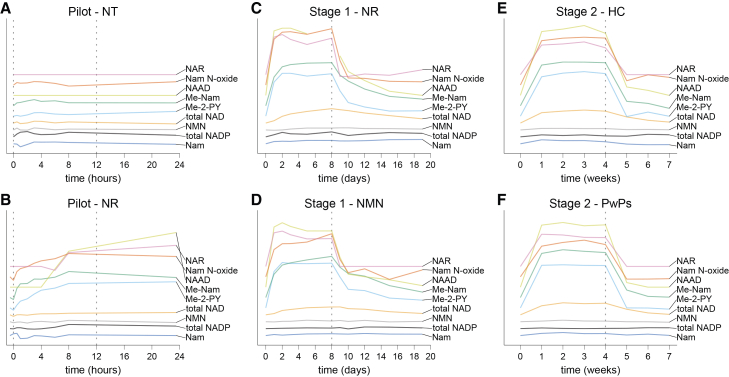
Comparison of metabolite changes over time in pilot, stage 1 and stage 2 of the NAD-brain study The plots show the time course of changes in all metabolite levels for the pilot without (A) and with (B) NR supplementation stage 1 upon NR (C) or NMN (D) supplementation, and stage 2 in healthy controls (E) and persons with PD (PwPs, F). Relative metabolite levels were log-transformed and positioned relative to each other for visualization purposes and improved comparability. NR, nicotinamide riboside; NMN, nicotinamide mononucleotide; HC, healthy control; PwPs, persons with Parkinson’s disease; total NAD, total nicotinamide adenine dinucleotide; total NADP, total nicotinamide adenine dinucleotide phosphate; NAR, nicotinic acid riboside; NAAD, nicotinic acid adenine dinucleotide; Nam, nicotinamide; Me-Nam, 1-methyl nicotinamide; Me-2-PY, N1-methyl-2-pyridone-5-carboxamide; Nam N-oxide, nicotinamide N-oxide.

Several NAD related metabolites responded faster to treatment initiation and cessation than NAD itself (Figure 6). Acidic intermediates such as NAR and NAAD increased within hours of the first dose—before a detectable NAD elevation—supporting a model in which deamidation of Nam and NR to NA and NAR, respectively, by the gut microbiome contributes to systemic NAD biosynthesis.^49^ In contrast, Nam degradation products (Me-Nam, Me-2-PY, and Nam N-oxide) increased within 30 min, indicating that a partial uptake and metabolism of NR to Nam happens quickly. Importantly, potentially adverse metabolites such as Me-2-PY normalized within 1–2 days of treatment cessation, suggesting that NAD precursor supplementation does not lead to sustained accumulation of these species beyond the treatment period.

In conclusion, NAD-AT with oral NR or NMN in humans produces a slow but robust and sustained elevation of systemic and cerebral NAD that stabilizes after ∼2 weeks and declines with similar kinetics after cessation. The response is consistent across health and PD, independent of sex, and highly reproducible within individuals. These findings establish key PK principles for the rational design of future NAD-targeted interventions in neurological and age-related diseases.

### Limitations of the study

Some limitations should be considered when interpreting the findings of this study. First, the ^31^P-MRS, especially on a 3T MR scanner, has limited sensitivity for measuring brain NAD levels. This results in relatively low signal-to-noise ratio, high technical (in addition to biological) variability, and the inability to distinguish between NAD^+^ and NADH. Future studies may consider using higher field strength systems, such as 7T MR, or applying finer voxel grids to improve sensitivity and spatial resolution. Second, while we observed considerable interindividual variability in blood NAD levels, we did not account for potential effects of cell composition (e.g., proportion of red blood cells), which may, in theory, influence whole blood NAD concentration.

## Resource availability

### Lead contact

Further information and requests for resources should be directed to and will be fulfilled by the lead contact, Charalampos Tzoulis (charalampos.tzoulis@uib.no).

### Materials availability

This study did not generate new unique reagents.

### Data and code availability

The source data for Figures and Tables is available in Data S1. Any additional information not included here or in the supplementary information can be made available by the corresponding authors upon reasonable request. The MATLAB code required for calculation of the peak amplitudes in ^31^P-MRS data, including the chemical shift and linewidth for all metabolites, and the R code required to reproduce the presented results have been deposited in the Neuromics Group repository https://git.app.uib.no/neuromics/nad-brain with no planned end date. Data can be accessed publicly by using the URL provided above.

## Acknowledgments

We are grateful to all the participants in the study. We thank Dr. Silje Skrede and Dr. Rolv Terje Lie for fruitful scientific discussion, and Therese Vetås and Gry-Hilde Nilsen for excellent technical assistance. The study was supported by grants from The Research Council of Norway (288164; C.T.), The KG Jebsen Foundation (SKGJ-MED-023; C.T.), The Western Norway Regional Health Authorities (C.T.), and Norway`s Parkinson Research Fund (C.D.). The funders had no role in study design, data collection, analysis, and interpretation.

## Author contributions

H.B. recruited and assessed study participants, performed analyses, analyzed and interpreted data, and drafted the manuscript. M.Svensen performed the ^31^P-MRS analyses, analyzed and interpreted data, and drafted the manuscript. H.E. participated in the study design, recruited and assessed study participants, analyzed and interpreted data, and drafted the manuscript. N.T. participated in the study design, recruited and assessed study participants, analyzed and interpreted data, and drafted the manuscript. E.V.S. participated in the study design, recruited and assessed study participants, and critically revised the manuscript. S.A.A.G. recruited and assessed study participants and critically revised the manuscript. M.Søgnen recruited and assessed study participants and critically revised the manuscript. V.S. performed the ^31^P-MRS scanning, interpreted data, and critically revised the manuscript. A.H. performed the ^31^P-MRS scanning, interpreted data, and critically revised the manuscript. G.-O.S. recruited and assessed study participants and critically revised the manuscript. Y.N.T.C. participated in the study conceptualization and acquisition of funding and critically revised the manuscript. G.S.N. participated in the data analysis and interpretation and critically revised the manuscript. F.R. participated in the study design, designed and applied scanning protocols, performed ^31^P-MRS analyses, analyzed and interpreted data, and drafted the manuscript. K.H. contributed to the conceptualization and design of the study, and to data collection. A.M. performed LC-MS/MS analyses. L.A performed LC-MS/MS analyses. O.Å. performed LC-MS/MS analyses. C.T. conceived, designed and directed the study, contributed to data analyses and interpretation, drafted the manuscript, and acquired funding for the study. C.D. conceived, designed and directed the study, contributed to data analyses and interpretation, drafted the manuscript, and acquired funding for the study. All authors have read and approved the manuscript.

## Declaration of interests

C.T. and C.D. are listed as inventors on international patent applications relating to the use of nicotinamide riboside as a treatment for Parkinson’s disease and related disorders. These patents have been filed by the Technology Transfer Office “Vestlandets Innovasjonsselskap AS (VIS)” on behalf of Haukeland University Hospital, Bergen, Norway (PCT/EP2022/067412, PCT/EP2023/060962, EP4284387).

## STAR★Methods

### Key resources table

**Table:** 

REAGENT or RESOURCE	SOURCE	IDENTIFIER
**Biological samples**		

Whole blood snap-frozen to -80°C at bedside	This paper	N/A

**Deposited data**		

Individual demographics data	This paper	N/A
Individual NADMed data	This paper	N/A
Individual 31P-MRS data	This paper	N/A
Individual LC-MS/MS data	This paper	N/A
R code used to analyze and present results	This paper	https://git.app.uib.no/neuromics/nad-brain
MatLab code required for calculation of peak amplitudes, chemical shift and linewidth for metabolites from ^31^P-MRS data	This paper	https://git.app.uib.no/neuromics/nad-brain

**Software and algorithms**		

3 T Siemens Biograph mMR PET/MR scanner, software version VE11P-SP03	Siemens Healthcare, Erlangen, Germany	N/A
Spectral Registration implementation from Gannet 3.0	Edden et al.^50^	https://github.com/richardedden/Gannet3.0; RRID:SCR_016049
OXSA v2.0.0 toolbox	Purvis et al.^51^	https://github.com/oxsatoolbox/oxsa
Matlab R2018b, v9.5	MathWorks, Natick, MA, USA	RRID:SCR_001622
R v4.4.2	R Foundation for Statistical Computing	https://cran.r-project.org; RRID:SCR_001905
Biorender	Biorender	https://www.biorender.com; RRID:SCR_018361

### Experimental model and study participant details

#### Trial design and oversight

NAD-brain was a phase I, single-center, open-label trial. The primary aim of the study was to establish the pharmacokinetic (PK) profile of NAD augmentation with oral NR and NMN. The trial was divided into three stages: a pilot study, stage 1 and stage 2. In the pilot, a single healthy male volunteer received NR or no intervention and was assessed for 24 hours, to explore the physiological fluctuation of blood and brain NAD levels and determine whether NAD levels would rise within 24 hours following the intake of an oral precursor (at time points 0 and 12 hours). Stage 1 was a crossover PK study with NR and NMN on six healthy volunteers (3 female/3 male). Stage 2 was a parallel group PK study with NR on six healthy volunteers (3 female/3 male) and six PwPs (3 female/3 male). Sex in this study refers to sex assigned at birth.

The trial was conducted at the Department of Neurology, Haukeland University Hospital, Norway. The pilot was conducted from 17.09.2022 to 18.09.2022, stage 1 from 29.01.2023 to 17.03.2023, and stage 2 from 08.01.2024 to 04.03.2024. Healthy individuals were recruited from the community at Haukeland University Hospital and the University of Bergen, Norway. PwPs were identified and recruited at the Department of Neurology, Haukeland University Hospital, Norway.

The trial was conducted in accordance with Good Clinical Practice (GCP) guidelines of the International Council for Harmonization, the principles of the Declaration of Helsinki, and Norwegian law. The protocol was approved by the Regional Committee for Medical and Health Research Ethics of Western Norway (ID: 496197). All participants provided written informed consent. No financial or other compensation was offered to participants. The trial was registered on www.Clinicaltrials.gov with identifier: NCT05698771.

#### Participants and eligibility

##### Healthy participants

Inclusion criteria for healthy individuals were: (i) Age 30-85 years at the time of enrollment. (ii) Neurologically healthy at the time of enrollment. Exclusion criteria for healthy individuals were: (i) History of acute or chronic neurological disorder affecting the central nervous system (CNS). Medical history of migraine, cluster headache, and tension headache was allowed, but not active attacks on the day of the study visits; (ii) Impaired renal function; (iii) Impaired hepatic function; (iv) Severe hematological disease; (v) Any psychiatric disorder that would interfere with compliance in the study; (vi) Any severe somatic illness that would make the individual unable to comply and participate in the study; (vii) Mitochondrial disease; (viii) Use of high dose vitamin B3 supplementation within 30 days of enrolment.

##### Persons with PD (PwPs)

Inclusion criteria for PwPs were: (i) clinical diagnosis of idiopathic PD according to the MDS criteria^52^; (ii) Hoehn and Yahr score <4 at time of enrollment,^53^ (iii) 123I-Ioflupane dopamine transporter imaging (DAT-scan) confirming nigrostriatal degeneration, (iv) Age 30-85 years at the time of enrollment.

Exclusion criteria for participants with PD were: (i) Dementia or other neurodegenerative disorders at baseline visit, (ii) Diagnosis of atypical parkinsonism (progressive supranuclear palsy, multiple system atrophy or corticobasal degeneration) or vascular parkinsonism; (iii) History of acute or chronic neurological disorder, other than PD, affecting the central nervous system (CNS). Medical history of migraine, cluster headache, and tension headache was allowed, but not active attacks on the day of the study visits; (iv) Impaired renal function; (v) Impaired hepatic function; (vi) Severe hematological disease; (vii) Any psychiatric disorder that would interfere with compliance in the study; (viii) Any severe somatic illness that would make the individual unable to comply and participate in the study; (ix) Mitochondrial disease; (x) Use of vitamin B3 supplementation within 30 days prior to enrollment.

### Method details

#### Procedures

The trial was divided into three stages: a pilot study, stage 1 and stage 2.

##### Pilot study

A healthy male participant was assessed in two sequential sessions on consecutive days of repeated blood sampling and ^31^phosphorus magnetic resonance spectroscopy (^31^P-MRS). The first session was without any intervention. The second session started the next day and involved oral supplementation with 600 mg NR at time points 0 and 12 hours. At each session, blood sampling and ^31^P-MRS were conducted nine times, at time points 0, 0.5, 1, 2, 3, 4, 6, 8, and 23.5 hours. The minimum possible time interval for combined blood sampling and ^31^P-MRS scanning was 30 min, determined by the practical execution of the MRS scan.

##### Stage 1

Six healthy participants (3 females, 3 males) completed a randomized, crossover trial with nicotinamide riboside (NR) and nicotinamide mononucleotide (NMN). Participants received 600 mg NR or 600 mg NMN twice daily (08:00 and 20:00 o`clock) for 8 days, followed by an 11-day washout period. A subsequent 9-day washout period was implemented to achieve a total washout of three weeks before crossing over to the alternate precursor for another 8-day supplementation period with an identical washout sequence. Blood sampling and ^31^P-MRS scans were performed during each supplementation phase at time points 0 (pre-dose), 1, 2, 3, 5, 8, 9, 10, 12, 15, and 19 days.

##### Stage 2

This stage involved 12 participants: 6 healthy individuals (3 females, 3 males) and 6 PwPs (3 females, 3 males). Participants received 600 mg NR twice daily (08:00 and 20:00 o`clock) for four weeks, followed by a three-week washout period. Blood sampling and ^31^P-MRS scans were performed at time points 0 (pre-dose), 1, 2, 3, 4, 5, 6, and 7 weeks. Sex was recorded as the biological sex. PwPs continued their standard anti-Parkinson medications without any modifications during the trial.

#### End points

The primary end point was the change of whole blood NAD levels (measured by LC-MS/MS and the NADMed assay, see details below and in the Study Protocol) and cerebral NAD levels (measured by ^31^P-MRS, see details below and in the Study Protocol) over time after the administration of oral NAD-AT. Secondary endpoints were interindividual differences and between-sex differences in the change in blood and cerebral NAD levels following the administration of oral NAD-AT.

#### Investigational compounds

Nicotinamide riboside chloride (NR; Tru Niagen, ChromaDex) and nicotinamide mononucleotide (NMN; NMN PRO300, ProHealth Longevity) capsules containing 300 mg NRCl or 150 mg NMN, respectively, were purchased via online providers. Drug compliance was determined by self-reporting from participants at study visits and a pill count of remaining medication at the end of the study. Compliance was 100 % in all stages for all individuals except for one individual in stage 2 with 98.2% due to one missed dose. NR and NMN were administered twice daily.

#### Blood sampling

Flash frozen whole blood samples were obtained by collecting blood in EDTA tubes and aliquoting into 250 μl aliquots. The blood samples were snap-frozen in liquid nitrogen precisely 2 min after blood was drawn.

#### NAD metabolite analysis from frozen whole blood

##### LC-MS/MS analysis

Targeted profiling of the NAD metabolome was performed at Bevital AS, Bergen, Norway (http://bevital.no).

The precipitation solution was prepared by mixing 6 ml ice-cold acetonitrile, 2 ml ice-cold methanol, 2 ml millipore ultrapure H_2_O and 10 μl of 0.1 % (10 μl) medronic acid. The solution was vortexed for 20 sec, before isotope labelled internal standards (ISTD) were added, and the solution vortexed again for 20 sec.

Whole blood samples were thawed in a water bath at 4 °C for 20 min and vortexed (time dependent on tube type and sample volume). 50 μl of thawed sample were added to 450ul of precipitation solution containing ISTD, vortexed for 15 sec, foil sealed and centrifuged for 10 min at 13000 xg. 100 μl supernatant were then transferred to 96-well plates, and further centrifuged for 5 min. The plate was then foiled and put in the autosampler.

The NAD metabolome profile was quantified by liquid chromatography-tandem mass spectrometry (LC-MS/MS) using an Agilent 1290 Infinity II LC System coupled with a Sciex 5500 Triple Quad Mass Spectrometer. RF-tuning was optimized using the instrument’s standardized quadrupole dipping procedure. Deproteinized whole blood (7μl) was injected into a XBridge BEH Amide XP Column (2.5 μm, 3.0 x 150 mm) with guard column. The guard and analytical columns were mounted in a column compartment set at 40 °C. The mobile phase consisted of two components, solution A: 80 % H_2_O, 20 % acetonitrile, 16 mM ammonium acetate, 5 μM medronic acid and solution B: 80 % acetonitrile, 20 % H_2_O, 4 mM ammonium acetate, 5 μM medronic acid. Samples were injected every 17.5 min, and analytes eluted at a flow rate of 250 μL/min. All gradient steps were linear. Method parameters were optimized as follows: ion-spray (5500 V), curtain gas (20 psig), collision gas (medium), ion source temperature (600 °C, and ion source gas 1 and 2 (40 psig). The analytes were detected in positive-ion multiple-reaction monitoring (MRM) mode with unit resolution at Q1 and Q3. An overview of the relevant MRM parameters for the analytes and internal standards is provided in Tables S2 and S3, respectively.

Limit of detection (LOD) was determined for each metabolite individually and ranged from 0.002-0.25 μmol/L. Within- and between-day coefficient of variation for each of the metabolites in the method ranged from 1-10 % and 2-11 %, respectively. For datapoints below the LOD, values of half LOD (LOD/2) were plotted.

#### NADMed assay

Additional analyses on whole blood were carried out by NADMed (Helsinki, Finland; www.nadmed.com). NAD metabolites (NAD^+^, NADH) were extracted from frozen blood in a single step using a proprietary extraction procedure, and each metabolite was measured individually using optimized cyclic enzymatic assays with colorimetric detection.

The NADMed assay is a validated alternative with performance characteristics in line with those of MS (for the relevant metabolites).^35^ For stability, the NADMed extraction method utilizes an extraction solution representing a non-buffer complex mixture of organic solvents in water. Samples are injected into a pre-warmed extraction solution to force protein unfolding and release non-covalently bound metabolites into solution. All pH and redox sensitive metabolites remain stable. Normalization of measured values is done per volume, corrected for dilutions during the extraction step and represented in μM concentration units in whole blood. Experimental conditions for redox profiling and data on method validation are presented elsewhere.^35^ Due to the lack of NADH data points from a few samples in these analyses, NAD^+^ data is reported for whole blood samples, which can, however, be considered as good representative of the total NAD pool.

#### NAD half-life determination

NAD half-life estimation was carried out assuming a first order reaction and using a one-phase exponential decay equation (t_1/2_ = 0.693/k) to the measured values of NAD concentration from the time point of highest NAD concentration (c_max_) to end of the washout period in stage 1. For optimal fitting, the following values were used: for NR: day 8, 9, 10, 12, 15 and 19; for NMN: day 9, 10, 12, 15 and 19. Analyses were carried out using the non-linear regression function for a one phase decay with default settings in GraphPad Prism version 6.07.

#### Magnetic resonance imaging and spectroscopy (MRI/S)

##### Acquisition

^31^P-MRS and analysis were carried out as previously described.^25^ Subjects were scanned using a 3 Tesla Biograph mMR PET/MR scanner (Siemens Healthcare, Erlangen, Germany) equipped with a commercially available double-resonant transmit/receive ^1^H/^31^P volume head coil (RAPID Biomedical, Würzburg, Germany) which allows for ^31^P-MR acquisitions. An anatomical T1-weighted image was acquired for positioning the volume of interest (VOI) within which ^31^P-MRS using a 3D chemical shift imaging (CSI) sequence was performed with WALTZ4-^1^H-decoupling and nuclear Overhauser effect (NOE) enhancement.^54^ The VOI was 240 mm x 240 mm x 80 mm, comprised of an 8 x 8 grid of voxels 30 mm x 30mm x 80 mm in size. This VOI was centered on the brain midline and aligned parallel to the posterior and anterior commissure for each subject by a trained radiographer. At 3 Tesla, the applied voxel size is required as the NAD signals overlap significantly with that of ATP-α and have low amplitudes. Thus, averaging the ^31^P-MRS signal over larger volumes rather than what is typically done in 3D CSI MRS was required for adequate NAD detection.

##### Spectral analysis

All processing and evaluation of spectra were performed in MATLAB R2018b (the MathWorks, Natick, MA). Signal phasing and subject motion was addressed with a modified version of the spectral registration algorithm from the Gannet MRS toolbox as previously described.^25^^,^^50^ The MatLab code required for the calculation of the peak amplitudes, including the chemical shift and linewidth for all metabolites, is available in the dedicated repository (see data and code availability).

Spectral post-processing and analysis was performed using the OXSA v2.0.0 toolbox,^51^ which fits the MRS signal to composite peaks using the AMARES algorithm.^55^ Signals were averaged over the whole occipital lobe, rejecting signals based on SNR, visual inspections as well as SNR, and visual inspections as well as Cramér-Rao Lower Bounds. A >30 % CRLB cutoff was used to reject signals that did not have a discernable NAD^+^ peak. The following metabolites were fitted: adenosine triphosphate (ATP) represented by all three phosphate groups (ATP-α, -β, and -γ), nicotinamide adenine dinucleotide (reported as total NAD, comprising both NAD^+^ and NADH), phosphocreatine (PCr), phosphocholine (PC) and glycerophosphocholine (GPC), phosphoethanolamine (PE) and glycerophosphoethanolamine (GPE), inorganic phosphate (Pi), membrane phospholipids (MP), and 2,3-diphosphoglycerate (2,3-DPG). In line with previously reported results and to account for subject-specific metabolic differences, we report NADtotal/ATP-alpha.^25^

### Quantification and statistical analysis

#### Sample size

A total of n = 12 participants (n = 6 healthy, n = 6 with PD) were included, in line with typical phase I PK studies. Based on prior data,^24^^,^^25^^,^^33^ this sample size was deemed sufficient to explore the time-dependent behavior of NAD levels in blood and brain following oral administration of NR or NMN, and to inform future trial design, while balancing feasibility and the participant procedure burden. The graphical abstract was created in BioRender: Berven, H. (2025) https://BioRender.com/nj6fgbz.

#### Statistical analysis

Descriptive statistics are presented as “mean ± standard deviation” for continuous variables. Mean percent change was calculated as the mean of individual percent change per visit per subject compared |to baseline. For LC-MS/MS datapoints below the LOD, values of half LOD (LOD/2) were generated for plotting metabolite profiles. Linear mixed models were used to analyze change over time between baseline and the end of the supplementation period and to analyze change from end of supplementation to end of monitoring period. For comparison of the change within groups the dependent variable was metabolite levels as a function of time with individual subjects as random effects. For the comparison between groups, the dependent variable was metabolite levels as a function of the interaction between time and treatment group with individuals as random effects. For the comparison between sexes within each group, the dependent variable was metabolite levels as a function of the interaction of time and sex, with individual subjects as random effects. Comparison of two time points was performed using paired two-tailed t-tests for normally distributed data and paired two-tailed Wilcoxon tests for non-normally distributed data. Normality was assessed using the Shapiro-Wilk test. Correlation was calculated using Spearman correlation. Intraclass correlation coefficients (ICC) estimates and their 95% confident intervals were calculated based on a single rating, consistency-agreement, 2-way mixed-effects model. All statistical analyses were carried out by the use of R (https://cran.r-project.org, version 4.4.2, R Foundation for Statistical Computing, Vienna, Austria) using the following packages; tidyverse version 2.0.0, ggpubr version 0.6.0, readxl version 1.4.3, cowplot version 1.1.3, nlme version 3.1.166, irr version 0.84.1, openxlsx version 4.2.7.1, ggrepel version 0.9.6.

### Additional resources

The NAD-brain study is registered on ww.Clinicaltrials.gov with the identifier: NCT05698771.

## Supplemental information

Document S1. Figures S1–S12 and Tables S1–S3

Data S1. Source data for figures and tables, related to Figures 1–6, S1–S12, Tables 1, 2, and S1

Data S2. Individual demographics data, related to Table 1

Data S3. Individual LC-MS/MS data, related to Figures 1–6, S1–S4, S6–S12, and Table 2

Data S4. Individual 31P-MRS data, related to Figures 1–4 and S2, and Table 2

Data S5. Individual NADMed data, related to Figures S5 and S6
