# Supplementary material for: The NAD-brain pharmacokinetic study of NAD augmentation in blood and brain using oral precursor supplementation
Source: iScience. 2026 Jan 27;29(3):114764. doi: 10.1016/j.isci.2026.114764 (PMC12996706; doi:10.1016/j.isci.2026.114764)
Supplement: Document S1. Figures S1–S12 and Tables S1–S3 [file mmc1.pdf]

iScience, Volume *29*

Supplemental information

The NAD-brain pharmacokinetic study of NAD augmentation in blood and brain using oral precursor supplementation

Haakon Berven, Magnus Svensen, Heidi Eikeland, Nora Tvedten, Erika V. Sheard, Solveig Amdahl Af Geijerstam, Mona Søgnen, Adrian McCann, Lena Arnsten, Ove Årseth, Vivian Skjeie, Arve Hjellbrekke, Geir-Olve Skeie, Yamila N. Torres Cleuren, Gonzalo S. Nido, Kristoffer Haugarvoll, Frank Riemer, Charalampos Tzoulis, and Christian Dölle

Supplemental information

Table S1: Adverse events during stage 1 and stage 2.

Stage 1	NR (n = 6)	NMN (n = 6)	Causal relationship to study drug (n)	Grade[a]
URTI	2	1	Unlikely (3)	Grade 1 (3)
Gastroenteritis	0	1	Unlikely (1)	Grade 1 (1)
**Stage 2**	**HC (n = 6)**	**PwPs (n = 6)**	**Causal relationship to study drug (n)**	**Grade[a]**
URTI	2	5	Unlikely (7)	Grade 1 (7)
Headache	0	1	Unlikely (1)	Grade 1 (1)
Viral laryngitis	1	0	Unlikely (1)	Grade 1 (1)
Herpes lingualis reactivation	0	1	Unlikely (1)	Grade 1 (1)
SARS-CoV-2[b]	0	1	Unrelated (1)	Grade 1 (1)
Fall with minor head trauma	0	1	Unlikely (1)	Grade 2 (1)

[a]Graded by increasing severity 1-3 in the following manner: Grade 1: Mild: An event that is easily tolerated by the participant, causing minimal discomfort and not interfering with everyday activities. Grade 2: Moderate: An event that causes sufficient discomfort to interfere with normal everyday activities. Grade 3: Severe: An event that prevents normal everyday activities. [b]Onset one week prior to baseline visit. PwPs: Persons with Parkinson's disease, HC: Healthy controls, URTI: Upper respiratory tract infection, SARS-CoV-2: Severe acute respiratory syndrome coronavirus 2. NR: Nicotinamide Riboside, NMN: Nicotinamide mononucleotide.

Table S2: Retention times and instrument settings for the measurement of the NAD metabolome in whole blood by LC-MS/MS. Related to STAR methods.

		T_r	Transition ions (*m/z*)		DP	EP	CE	CXP	
**Analyte**	**Abbreviation**	**(min)**	**Precursor**	**Product**	**(V)**	**(V)**	**(V)**	**(V)**	**Mode**
Nicotinamide	Nam	2.1	124.0	80.9	65	8	30	14	Positive
N-methyl-2-pyridone-5-carboxamide	Me-2-PY	2.3	153.2	110.3	70	9	30	10	Positive
Nicotinamide N-oxide	Nam-N-oxide	2.9	139.0	106.0	50	5	27	10	Positive
Nicotinic acid	NA	3	124.0	80.0	70	7	31	9	Positive
N-methyl-4-pyridone-3-carboxamide	Me-4-PY	3.6	153.2	92.1	60	8	35	9	Positive
N1-methylnicotinamide	Me-Nam	5.3	137.3	94.0	65	8	30	9	Positive
Nicotinic acid riboside	NAR	5.4	256.0	124.0	50	8	17	11	Positive
Nicotinamide riboside	NR	6.2	255.1	123.0	60	10	20	13	Positive
Nicotinamide adenine dinucleotide (reduced)	NADH	7.3	666.0	649.0	119	10	20	11	Positive
Nicotinamide adenine dinucleotide (oxidized)	NAD^+	9	664.0	136.1	200	10	45	10	Positive
Nicotinic acid adenine dinucleotide	NAAD	9.8	665.0	523.9	120	10	24	10	Positive
Nicotinamide mononucleotide	NMN	9.9	335.1	122.6	70	10	20	10	Positive
Nicotinamide adenine dinucleotide phosphate (reduced)	NADPH	11	746.1	729.0	119	10	20	11	Positive
Nicotinamide adenine dinucleotide phosphate (oxidized)	$NADP^+$	12.4	744.1	603.8	220	10	30	20	Positive

T_r: Retention time, DP: Declustering potential, EP: Entrance potential, CE: Collision energy, CXP: Collision cell exit potential.

Table S3: Retention times, concentrations and instrument settings for the isotope labeled internal standards. Related to STAR methods.

Analyte	Abbreviation	[] µmol/L	T_r (min)	Transition ions (*m/z*) Precursor	Product	DP (V)	EP (V)	CE (V)	CXP (V)	Mode
Nicotinamide-2,4,5,6-d4	Nam-d4[a]	26.242	2.1	128.0	84.9	65	8	30	14	Positive
Nudifloramide-D3	Me-2-PY-d3	5.000	2.3	156.2	113.3	110	10	30	22	Positive
Nicotinamide-d4 N-oxide (d4-major)	Nam N-oxide-d4	0.330	2.9	143.0	110.0	50	5	27	10	Positive
Nicotinic-d4 acid	NA-d4	0.100	3	128.0	84.0	70	7	31	9	Positive
N-Methyl-4-pyridone-3-carboxamide-d3	Me-4-PY-d3	0.400	3.6	156.2	95.1	60	8	35	9	Positive
N-Methylnicotinamide-d4 (d4-major)	Me-Nam-d4	0.560	5.3	141.3	98.0	65	8	30	9	Positive
Nicotinic acid-d4 riboside	NAR-d4	0.110	5.4	260.0	128.0	50	8	17	11	Positive
Nicotinamide riboside-d4 trifluoromethanesulfonate (d3-major), α/β mixture	NR-d4	0.110	6.2	259.1	127.0	60	10	20	13	Positive
β-Nicotinamide adenine dinucleotide-d4, reduced form, diammonium salt	NADH-d4	1.000	7.3	670.0	653.0	120	10	24	11	Positive
β-Nicotinamide adenine dinucleotide-d4 (oxidized form)	NAD^+-d4[a]	4.490	9	668.0	136.1	200	10	45	10	Positive
β-Nicotinamide-d4 mononucleotide (d4-major)	NMN-d4[a]	0.700	9.9	339.1	126.6	70	10	20	10	Positive

[a]In the absence of authentic isotope-labelled internal standards for NAAD, NADPH, and $NADP^+$, the internal standards NAM-d4, NAD-d4, and NMN-d4 were used as surrogate internal standards. []: Final internal standard concentration, T_r: Retention time, DP: Declustering potential, EP: Entrance potential, CE: Collision energy, CXP: Collision cell exit potential.

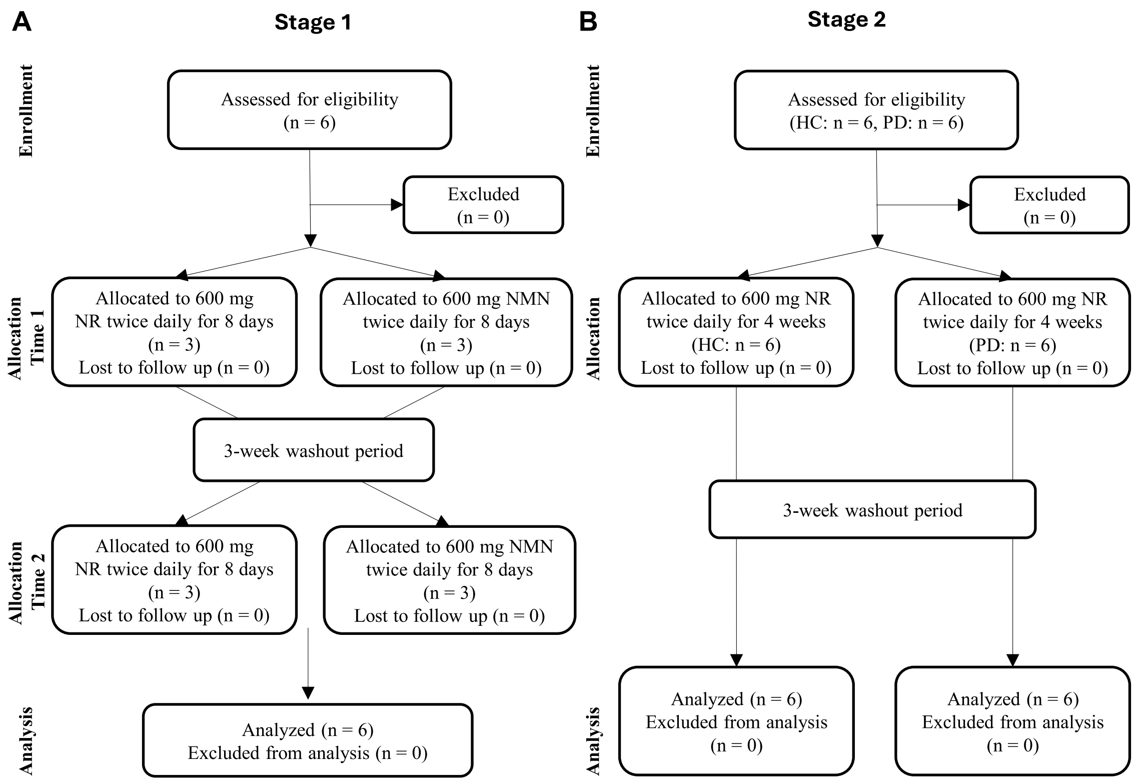

Figure S1. CONSORT diagram.

CONSORT diagram illustrating the study design and progress of the study for (**A**) stage 1 and (**B**) stage 2.

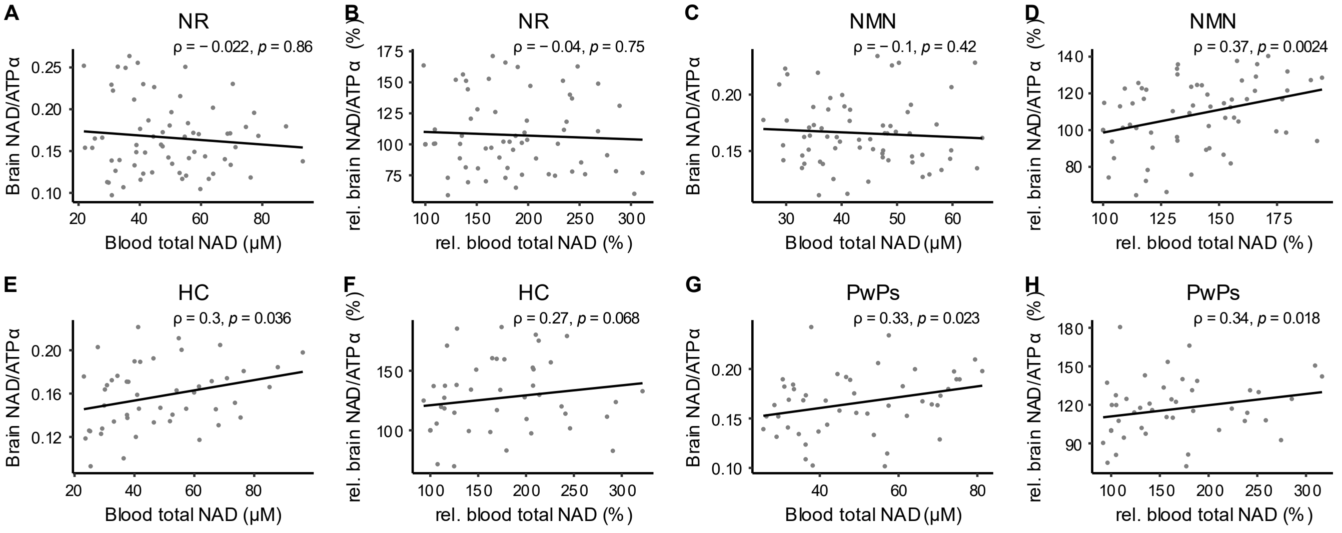

Figure S2. Correlation of blood total NAD to brain NAD/ATPα levels. Related to Figure 2 and 3.

Correlation between blood total NAD and brain NAD/ATPα levels. Top row (**A-D**) shows values from stage 1 with NR (**A**, **B**; n=6) and NMN (**C**, **D**; n=6) supplementation, bottom row (**E-H**) from stage 2 for healthy controls (**E**, **F**; n=6) and persons with PD (**G**, **H**; n=6). Plots show absolute (**A**, **C**, **E**, **G**) and relative (**B**, **D**, **F**, **H**) values. Coefficients and p-values shown calculated using Spearman correlation. NR: Nicotinamide riboside, NMN: Nicotinamide mononucleotide, HC: Healthy controls, PwPs: Persons with Parkinson's disease, total NAD: Total nicotinamide adenine dinucleotide, ATP: Adenosine triphosphate.

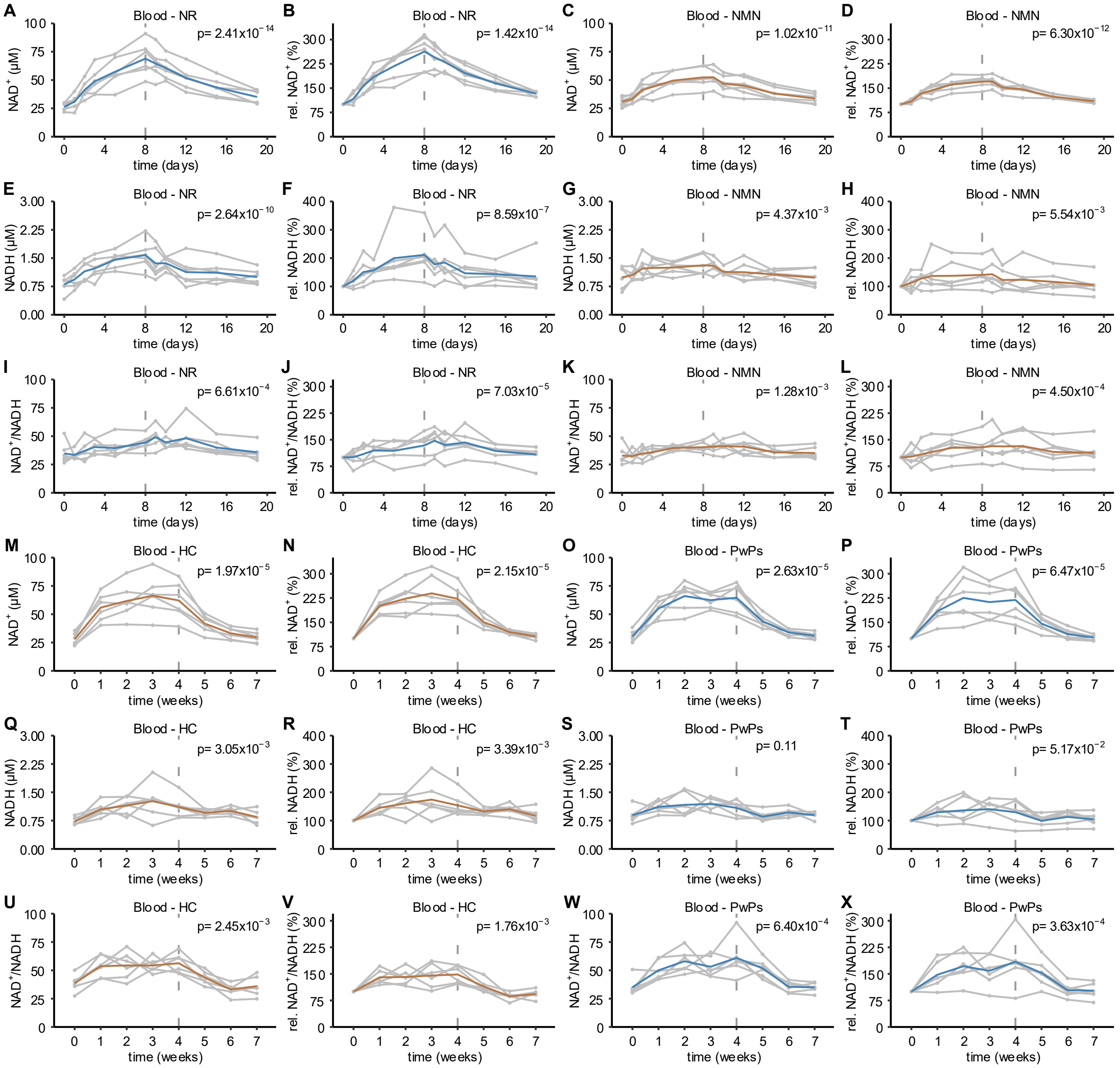

Figure S3. Changes in NAD^+, NADH and NAD^+/NADH in stage 1 and stage 2 of the NAD-brain study. Related to Figure 2 and 3.

The plots show absolute and relative data from stage 1(**A-L**) and stage 2 (**M-X**) for NAD^+ (**A-D**, **M-P**), NADH (**E-F**, **Q-T**) and NAD^+ / NADH ratio (**I-L**, **U-X**). Grey lines show individual curves. (**A-L**) Blue and brown lines show the mean after NR and NMN supplementation, respectively. (**M-X**) Blue and brown lines show the mean in healthy controls and persons with PD, respectively. n = 6 for each group. The dashed line indicates the last time point of oral NR and NMN supplementation. P-values represent statistical testing between baseline and the timepoint indicated by the dashed line. P-values were calculated using a linear mixed model where the dependent variable was metabolite level as a function of time with individual

participants as random effects. NAD^+: Nicotinamide adenine dinucleotide (oxidized), NADH: Nicotinamide adenine dinucleotide (reduced), NR: Nicotinamide riboside, NMN: Nicotinamide mononucleotide, HC: Healthy controls, PwPs: Persons with Parkinson's disease.

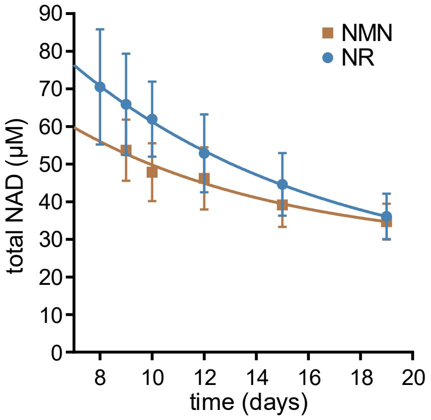

Figure S4. Estimation of half-life of elevated total NAD levels. Related to Figure 2.

For the estimation of the half-life of total NAD an exponential decay (first order reaction) was assumed. Data from time point of maximal total NAD levels (NR: day 8; NMN: day 9) until end of the washout period were included. Total NAD: Total nicotinamide adenine dinucleotide, NMN: Nicotinamide mononucleotide, NR: Nicotinamide riboside.

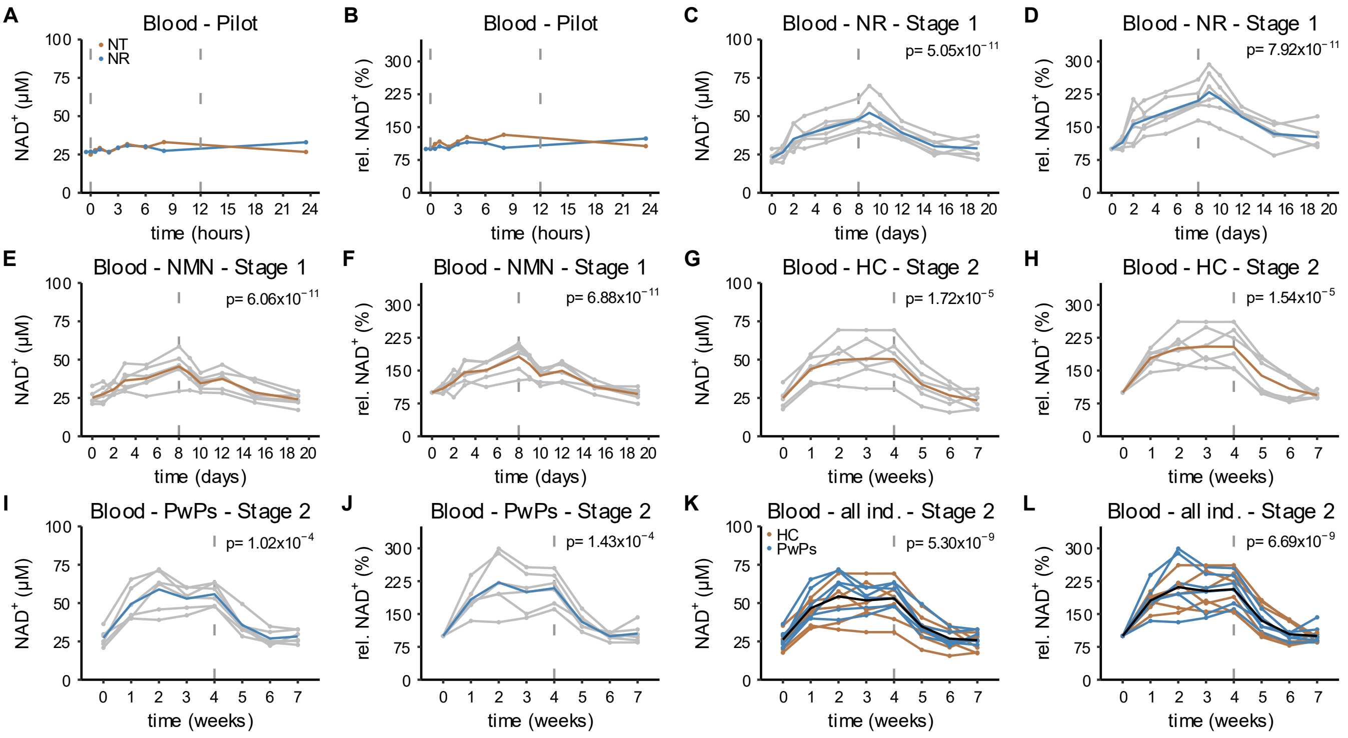

Figure S5. Changes in NAD$^+$ over time measured by the NADMed method. Related to Figure 1-3. Plots show changes of blood NAD$^+$ over time measured with the NADMed method. **A**, **B**) Absolute (**A**) and relative (**B**) NAD$^+$ levels measured during 24 hours in the pilot experiment (n=1). Brown curves represent no treatment, blue represents NR supplementation. **C-F**) Absolute (**C**, **E**) and relative (**D**, **F**) NAD$^+$ levels measured during stage 1 (n=6) with oral supplementation of NR (**C**, **D**) or NMN (**E**, **F**). Individuals' curves are shown in grey, means in blue and brown for NR and NMN supplementation, respectively. **G-L**) Absolute (**G**, **I**, **K**) and relative (**H**, **J**, **L**) whole blood NAD$^+$ levels measured during stage 2 in healthy controls (**G**, **H**; n=6), persons with PD (**I**, **J**; n=6) and all individuals combined (**K**, **L**). In (**E-J**), individuals' curves are shown in grey, and means in blue and brown for healthy controls and persons with PD, respectively. In **K**, **L**) individual curves are shown in blue and brown, and means in black. Dashed lines indicate time of NR supplementation (**A**, **B**) or last time point of oral NR and NMN supplementation (**C-L**). P-values represent statistical testing between baseline and the timepoint indicated by the dashed line. P-values calculated using a linear mixed model where the dependent variable was metabolite level as a function of time with individual participants as random effects. NT: no treatment, NAD$^+$: nicotinamide adenine dinucleotide (oxidized), NR: Nicotinamide riboside, NMN: Nicotinamide mononucleotide, HC: Healthy controls, PwPs: Persons with Parkinson's disease, ind.: Individuals.

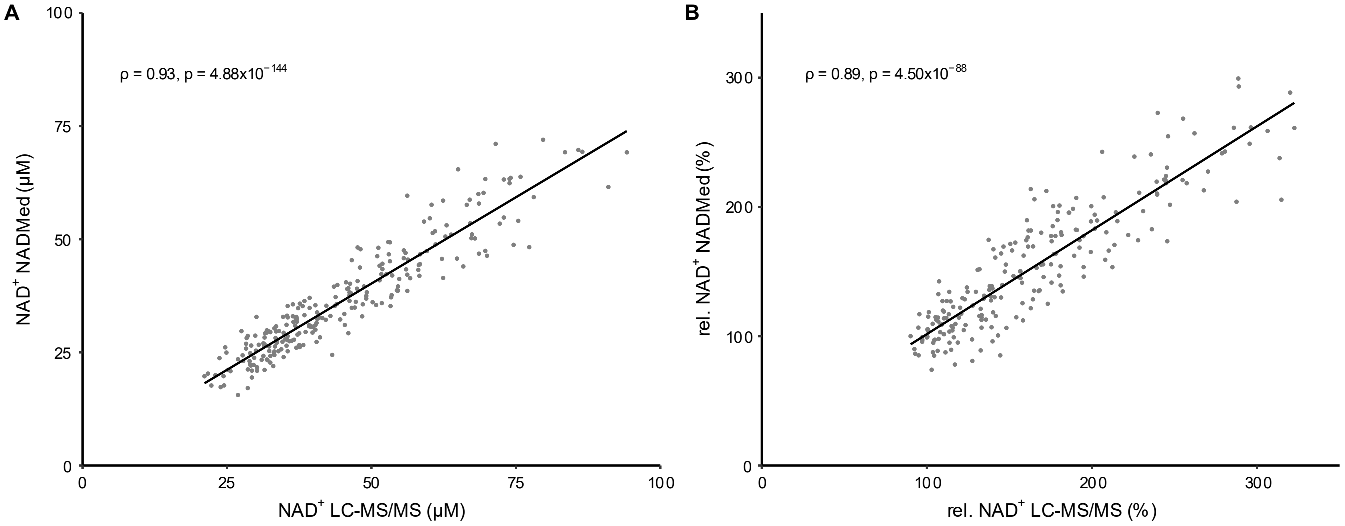

Figure S6. Correlation of NAD^+ measurements in blood between LC-MS/MS and NADMed methods. Related to Figure 1-3.

(**A**). Whole blood NAD^+ levels from all paired measurements in the study (n=247). (**B**) Whole blood relative NAD^+ levels compared to baseline from all paired measurements in the trial (n=247). Coefficients and p-values were calculated using Spearman correlation. NAD^+: Nicotinamide adenine dinucleotide (oxidized form).

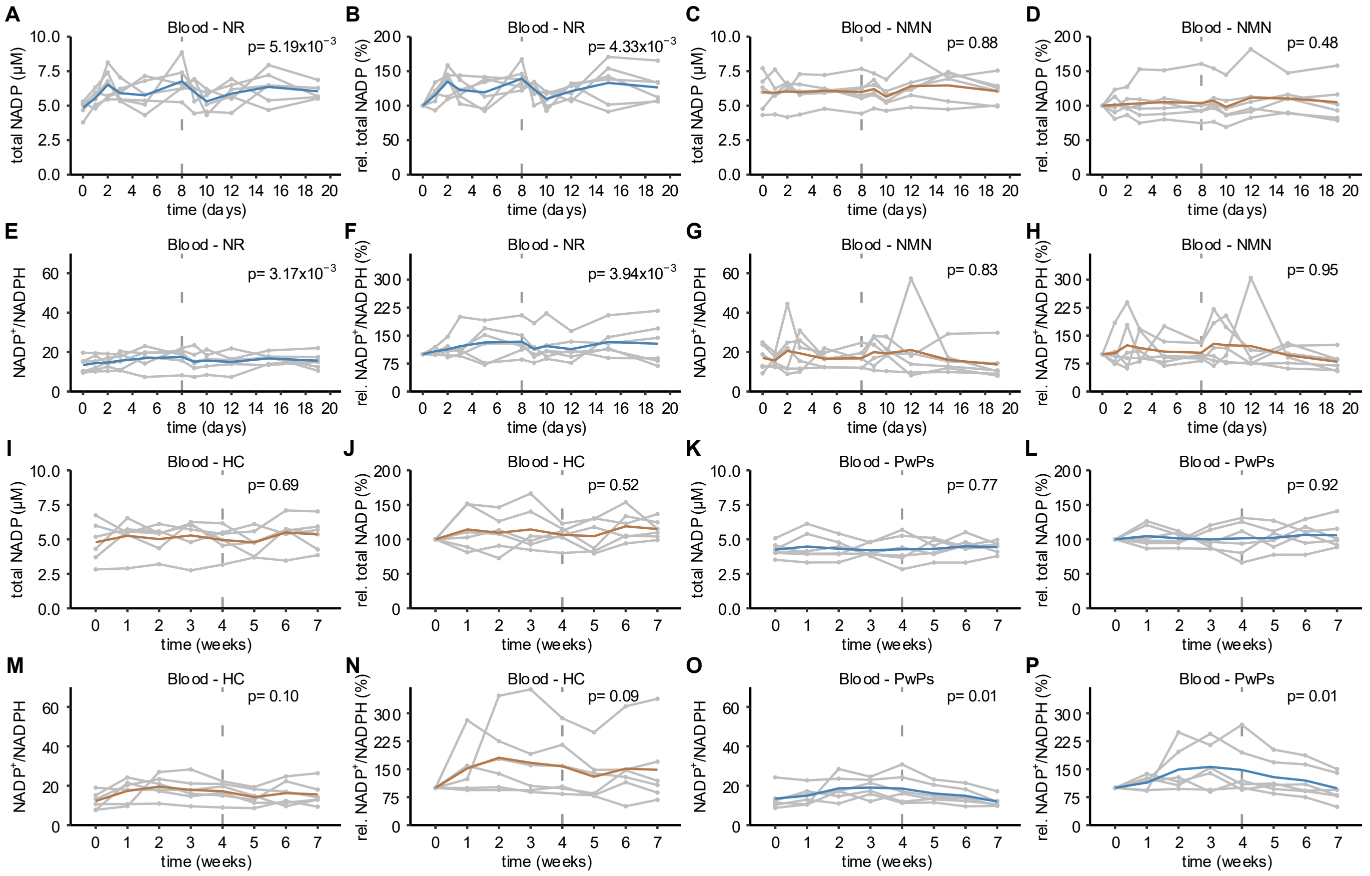

Figure S7. Change in total NADP and NADP$^+$/NADPH levels in stage 1 and stage 2 of the NAD-brain study. The plots show data from individuals in stage 1 (n=6) upon oral supplementation with NR or NMN (**A-H**), and from individuals in stage 2 (HC:n=6; PwPs: n=6) upon oral supplementation with NR (**I-P**). Grey lines show individual curves. Blue and brown lines show the mean with NR and NMN supplementation, respectively, in stage 1 (**A-H**), or in healthy controls and persons with PD in stage 2 (**I-P**). The dashed line indicates the last time point of oral NR and NMN supplementation. **A**, **C**, **I**, **K**) Total NADP levels in whole blood. **B**, **D**, **J**, **L**) Relative total NADP levels in whole blood compared to baseline. **E**, **G**, **M**, **O**) NADP$^+$/NADPH levels in whole blood. **F**, **H**, **N**, **P**) Relative NADP$^+$/NADPH levels in whole blood compared to baseline. P-values represent statistical testing between baseline and the timepoint indicated by the dashed line. P-values were calculated using a linear mixed model where the dependent variable was metabolite level as a function of time with individual participants as random effects. Total NADP: Total nicotinamide adenine dinucleotide phosphate, NADP$^+$: Nicotinamide adenine dinucleotide phosphate (oxidized), NADPH: Nicotinamide adenine dinucleotide phosphate (reduced), NR: Nicotinamide riboside, NMN: Nicotinamide mononucleotide, HC: Healthy controls, PwPs: Persons with Parkinson’s disease.

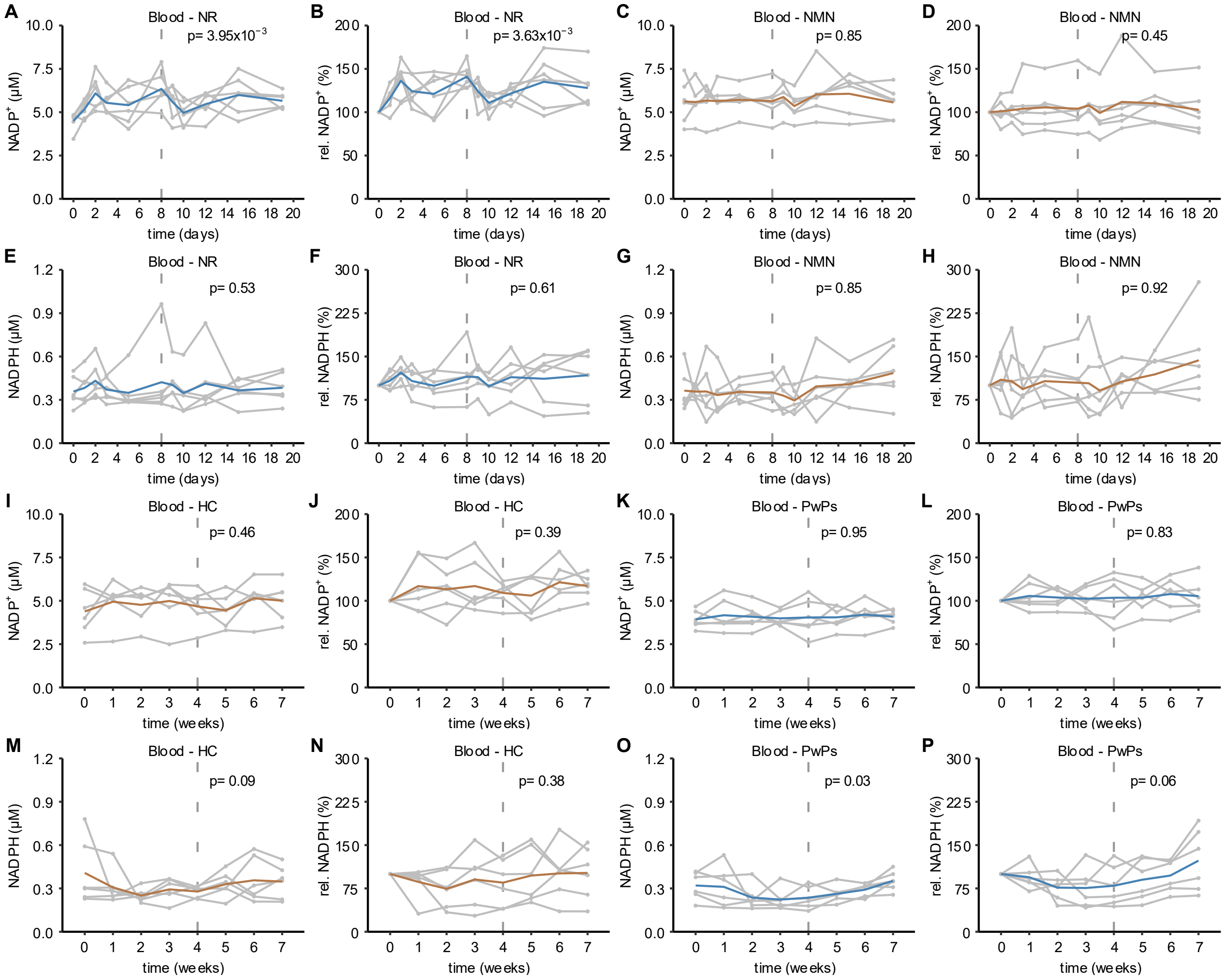

Figure S8. Change in NADP$^+$ and NADPH levels in stage 1 and stage 2 of the NAD-brain study. The plots show data from individuals in stage 1 (n=6) upon oral supplementation with NR or NMN (**A-H**), and from individuals in stage 2 (HC:n=6; PwPs: n=6) upon oral supplementation with NR (**I-P**). Grey lines show individual curves. Blue and brown lines show the mean with NR and NMN supplementation, respectively, in stage 1 (**A-H**), or in healthy controls and persons with PD in stage 2 (**I-P**). The dashed line indicates the last time point of oral NR and NMN supplementation. **A**, **C**, **I**, **K**) NADP$^+$ levels in whole blood. **B**, **D**, **J**, **L**) Relative NADP$^+$ levels in whole blood compared to baseline. **E**, **G**, **M**, **O**) NADPH levels in whole blood. **F**, **H**, **N**, **P**) Relative NADPH levels in whole blood compared to baseline. P-values represent statistical testing between baseline and the timepoint indicated by the dashed line. P-values were calculated using a linear mixed model where the dependent variable was metabolite level as a function of time with individual participants as random effects. NADP$^+$: Nicotinamide adenine dinucleotide phosphate (oxidized), NADPH:

Nicotinamide adenine dinucleotide phosphate (reduced), NR: Nicotinamide riboside, NMN: Nicotinamide mononucleotide, HC: Healthy controls, PwPs: Persons with Parkinson's disease.

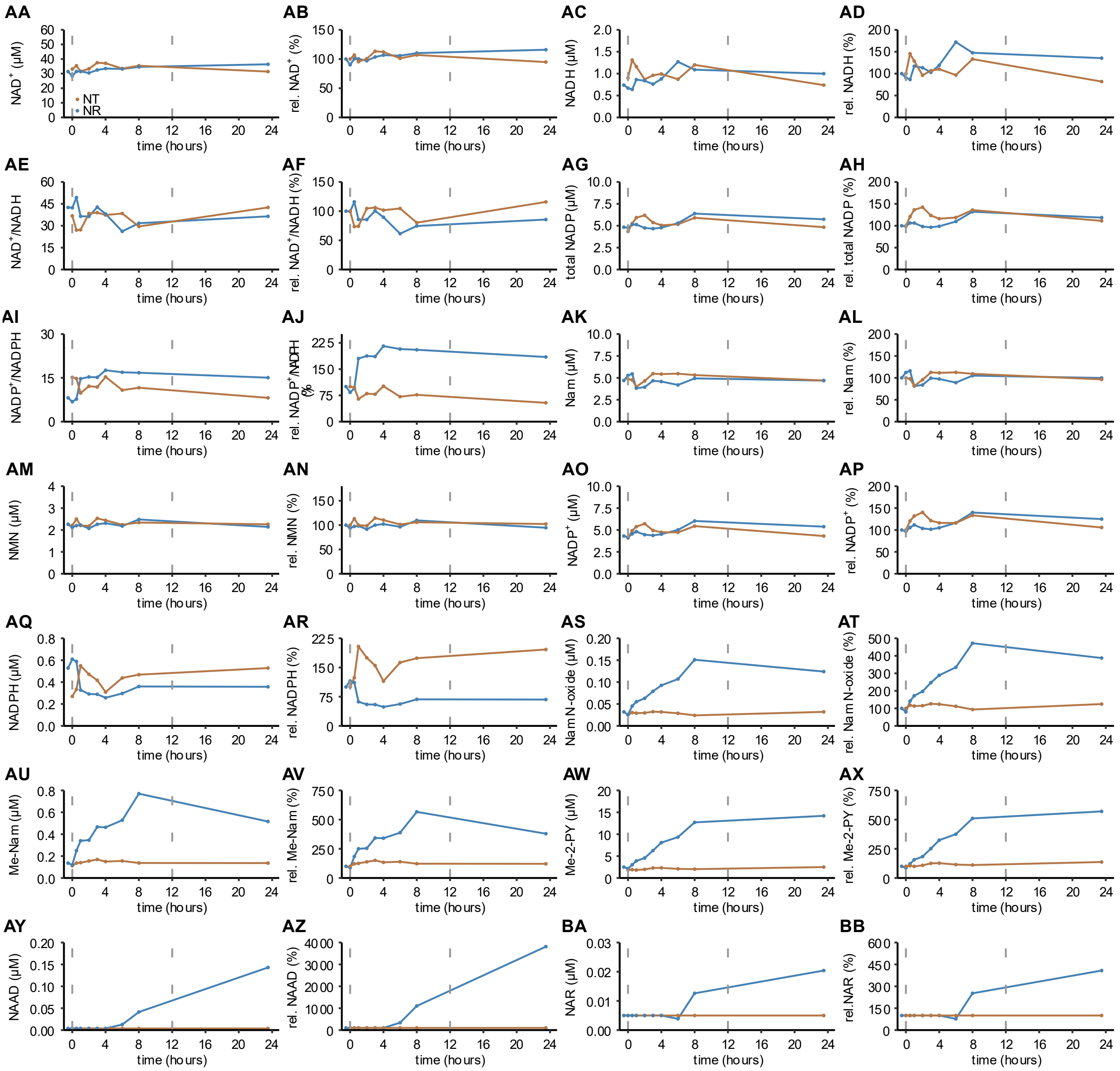

Figure S9. Changes in NAD and NAD-related metabolites during the pilot of the NAD-brain study. Related to Figure 5. One healthy male individual received 600 mg NR twice daily (NR) or not (NT) and was monitored over 24 hours. Metabolites levels from whole blood are shown as absolute levels (first and third columns) and relative levels compared to baseline (second and fourth columns). The dashed lines indicate time of NR supplementation. NR: Nicotinamide riboside, NT: No treatment, NAD$^+$: Nicotinamide adenine dinucleotide (oxidized), NADH: Nicotinamide adenine dinucleotide (reduced), NADP$^+$: Nicotinamide adenine dinucleotide phosphate (oxidized), NADPH: Nicotinamide adenine dinucleotide phosphate (reduced), Me-Nam: 1-methyl nicotinamide, NAAD: Nicotinic acid adenine dinucleotide, Me-2-PY: N1-methyl-2-pyridone-5-carboxamide, Nam: Nicotinamide, Nam N-

oxide: Nicotinamide N-oxide, NAR: Nicotinic acid riboside, NMN: Nicotinamide mononucleotide.

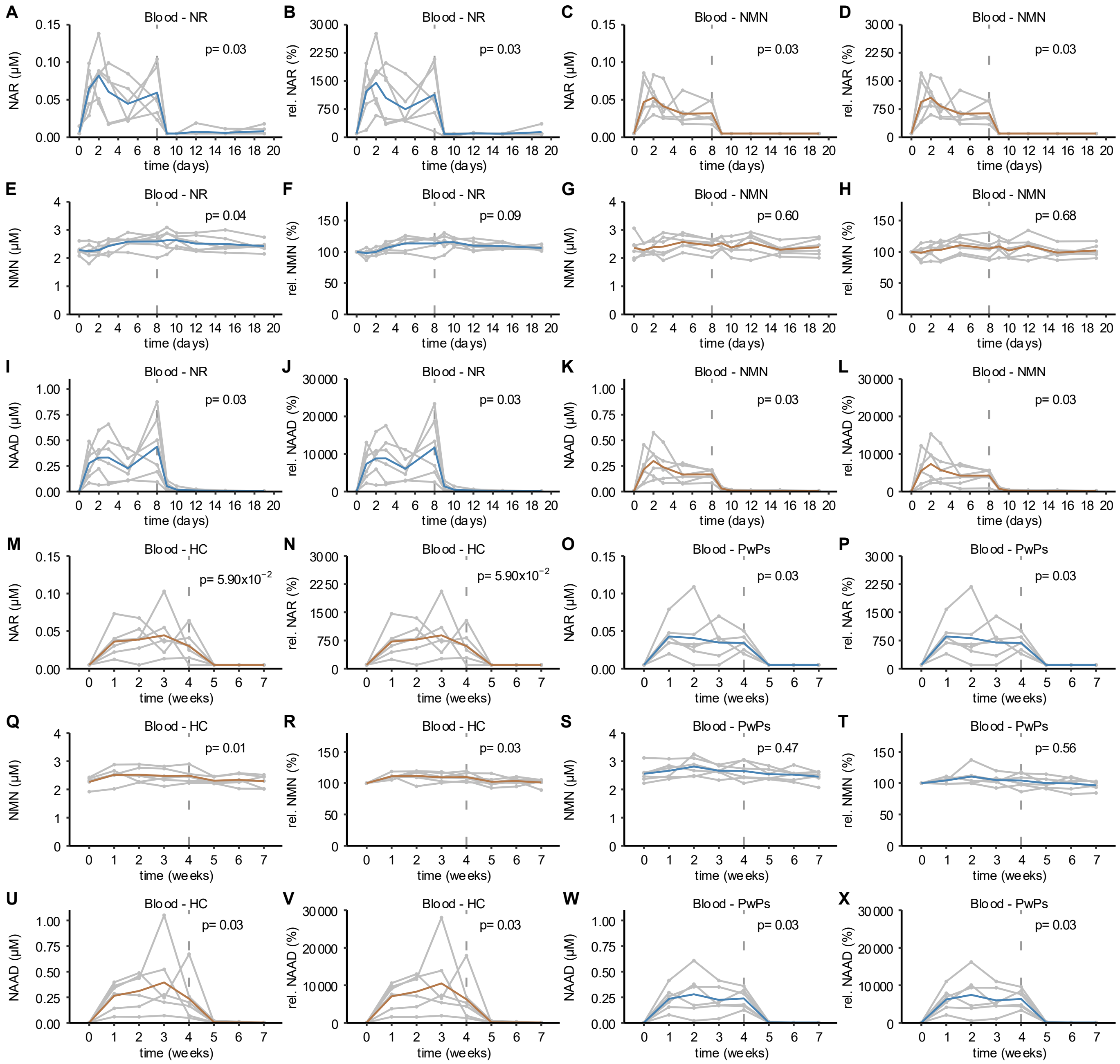

Figure S10: Changes in NAR, NMN and NAAD levels in stage 1 and stage 2 of the NAD-brain study. Related to Figure 5. The plots show data from whole blood of individuals in stage 1 (n=6) upon oral supplementation with NR or NMN (**A-L**), and from individuals in stage 2 (HC:n=6; PwPs: n=6) upon oral supplementation with NR (**M-X**). Grey lines show individual curves. Blue and brown lines show the mean with NR and NMN supplementation, respectively, in stage 1 (**A-L**), or in healthy controls and persons with PD in stage 2 (**M-X**). The dashed lines indicate the last time point of oral NR and NMN supplementation. **A**, **C**, **M**, **O**) absolute NAR levels. **B**, **D**, **N**, **P**) Relative NAR levels compared to baseline. **E**, **G**, **Q**, **S**) absolute NMN levels. **F**, **H**, **R**, **T**) Relative NMN levels compared to baseline. **I**, **K**, **U**, **W**) absolute NAAD levels. (**J**, **L**, **V**, **X**) Relative NAAD levels compared to baseline. The P-values represent statistical testing

within groups. All p-values were calculated using paired two-sided t-test or Wilcoxon tests between baseline and the last day of supplementation. NR: Nicotinamide riboside, NMN: Nicotinamide mononucleotide, NAR: Nicotinic acid riboside, NAAD: Nicotinic acid adenine dinucleotide, HC: Healthy controls, PwPs: Persons with Parkinson's disease.

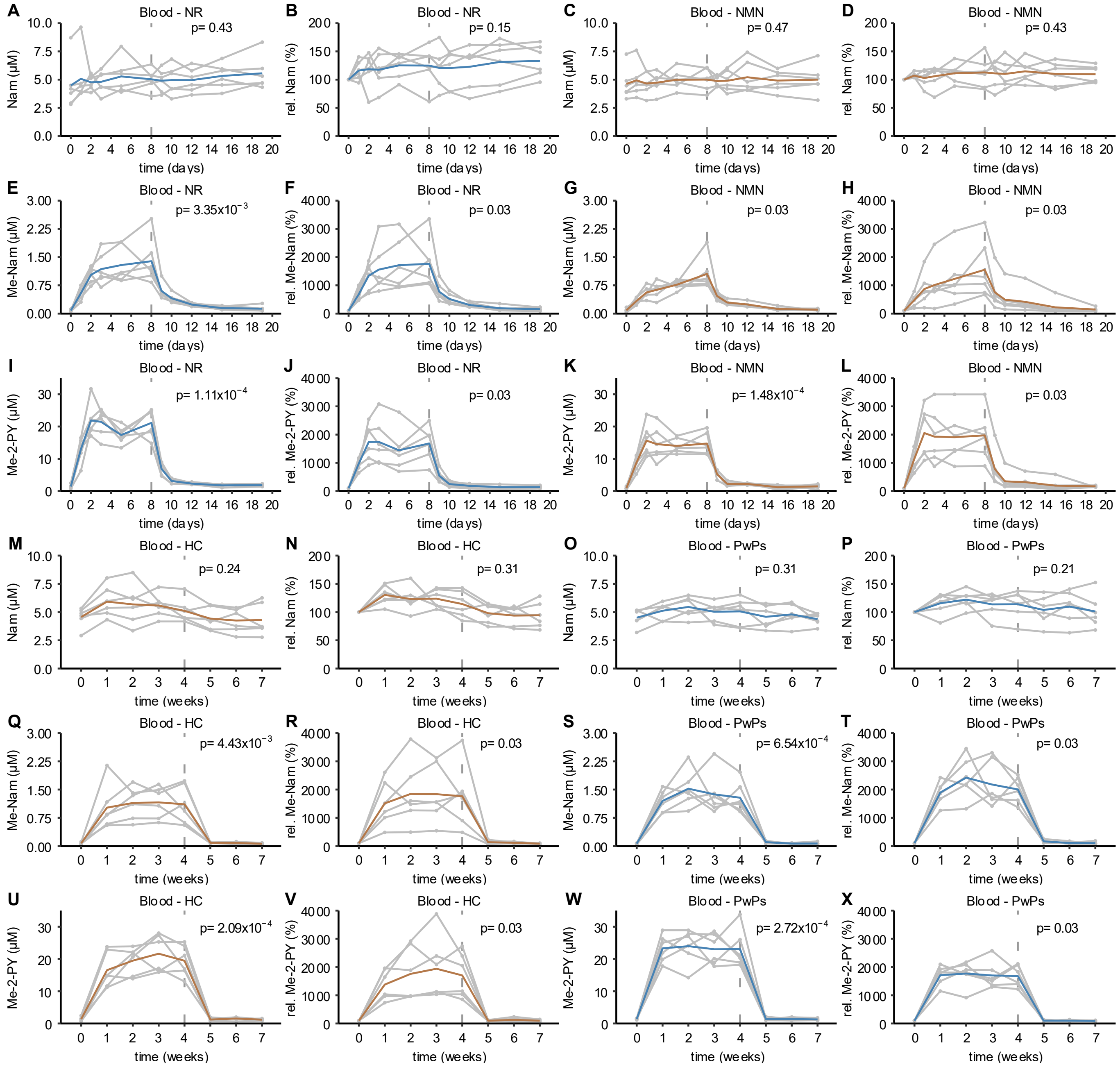

Figure S11: Change in Nam, Me-Nam and Me-2-PY levels in stage 1 and stage 2 of the NAD-brain study. Related to Figure 5. The plots show data from whole blood of individuals in stage 1 (n=6) upon oral supplementation with NR or NMN (**A-L**), and from individuals in stage 2 (HC:n=6; PwPs: n=6) upon oral supplementation with NR (**M-X**). Grey lines show individual curves. Blue and brown lines show the mean with NR and NMN supplementation, respectively, in stage 1 (**A-L**), or in healthy controls and persons with PD in stage 2 (**M-X**). The dashed lines indicate the last time point of oral NR and NMN supplementation. **A**, **C**, **M**, **O**) absolute Nam levels. **B**, **D**, **N**, **P**) Relative Nam levels compared to baseline. **E**, **G**, **Q**, **S**) absolute Me-Nam levels. **F**, **H**, **R**, **T**) Relative Me-Nam levels compared to baseline. **I**, **K**, **U**, **W**) absolute Me-2-PY levels. (**J**, **L**, **V**, **X**) Relative Me-2-PY levels compared to baseline. P-

values represent statistical testing within groups. All p-values were calculated using paired two-sided t-test or Wilcoxon tests between baseline and the last day of supplementation. NR: Nicotinamide riboside, NMN: Nicotinamide mononucleotide, HC: Healthy controls, PwPs: Persons with Parkinson’s disease, Nam: Nicotinamide, Me-Nam: 1-methyl nicotinamide, Me-2-PY: N1-methyl-2-pyridone-5-carboxamide.

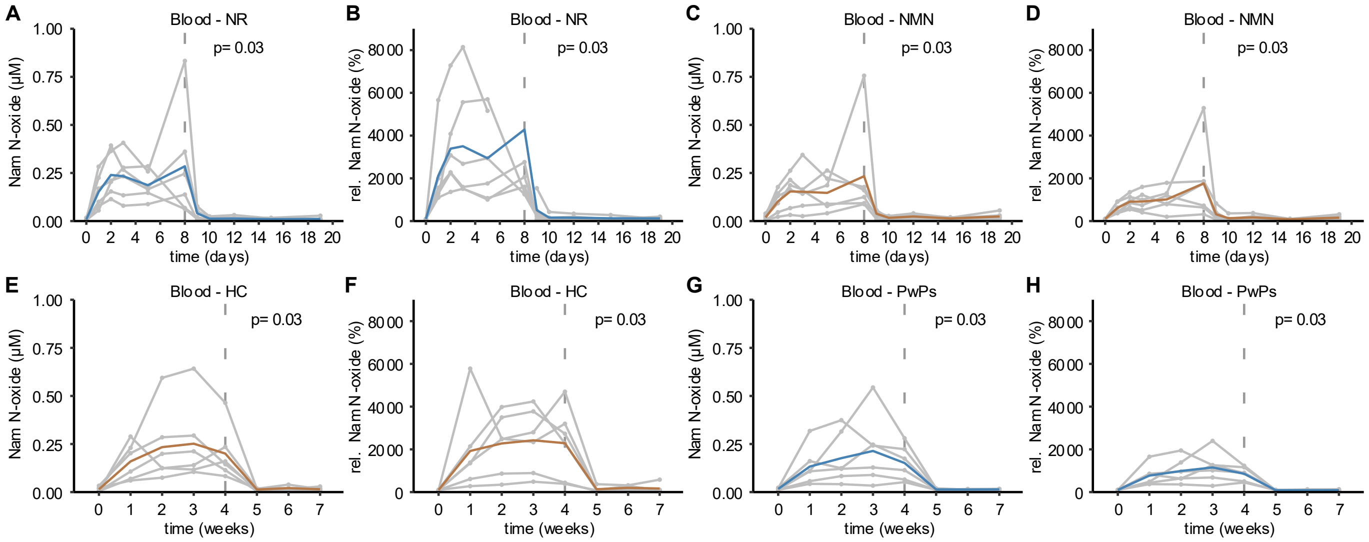

Figure S12: Change in Nam N-oxide levels in stage 1 and stage 2 of the NAD-brain study. Related to Figure 5.

The plots show data from whole blood of individuals in stage 1 (n=6) upon oral supplementation with NR or NMN (**A-D**), and from individuals in stage 2 (HC:n=6; PwPs: n=6) upon oral supplementation with NR (**E-H**). Grey lines show individual curves. Blue and brown lines show the mean with NR and NMN supplementation, respectively, in stage 1 (**A-D**), or in healthy controls and persons with PD in stage 2 (**E-H**). The dashed lines indicate the last time point of oral NR and NMN supplementation. **A**, **C, E, G, O**) absolute Nam N-oxide levels. **B**, **D**, **F**, **H**) Relative Nam N-oxide levels compared to baseline. P-values represent statistical testing within groups. All p-values were calculated using paired two-sided t-test or Wilcoxon tests between baseline and the last day of supplementation. NR: Nicotinamide riboside, NMN: Nicotinamide mononucleotide, HC: Healthy controls, PwPs: Persons with Parkinson's disease, Nam N-oxide: Nicotinamide N-oxide.
